# Views on mental health recovery in primary and community mental healthcare services in Thailand: A qualitative study

**DOI:** 10.1371/journal.pone.0353706

**Published:** 2026-07-20

**Authors:** Natthapon Inta, Annmarie Grealish, Mary Leamy

**Affiliations:** 1 Florence Nightingale Faculty of Nursing, Midwifery & Palliative Care, King’s College London, London, United Kingdom; 2 Princess Agrarajakumari Faculty of Nursing, Chulabhorn Royal Academy, Bangkok, Thailand; 3 School of Nursing and Midwifery, Health Research Institute, University of Limerick, Limerick, Ireland; Taipei Medical University, TAIWAN

## Abstract

**Introduction:**

Strong community-based services and healthcare providers with a clear understanding and positive attitudes towards recovery, are central to delivering good quality mental health care in collaboration with service users. Although recovery has been explored in several Asian contexts, its interpretation remains diverse, and consensus on the definition and meaning of personal recovery in Thailand is limited. Further research is needed to explore how mental health service users, carers, and healthcare professionals understand and experience recovery and recovery support, in order to advance recovery-oriented practice within the local sociocultural context.

**Aim:**

To explore thoughts, perceptions, and experiences of mental health service users, carers, and healthcare professionals on mental health recovery and recovery support in Thailand.

**Methods:**

A qualitative descriptive study using reflexive thematic analysis, which forms the experience gathering phase of an Experience Based Co-Design (EBCD) study. Thirty semi-structured interviews and two feedback workshops with three service users and eight healthcare professionals were conducted.

**Results:**

Four themes were identified: (i) conceptions of mental health recovery, (ii) attitudes towards recovery, (iii) characteristics of a successful recovery journey, and (iv) factors impeding recovery. Participants viewed recovery as beginning with clinical and functional improvement, progressing to personal recovery characterised by hope, meaning, and social connectedness. Family support, community acceptance, and supportive healthcare relationships were central to recovery, while barriers operated at individual, family, service, and community levels.

**Conclusion:**

This study integrates the perspectives of mental health service users, carers, and healthcare professionals to generate new insights into personal recovery within Thai community mental health care. From these findings, we propose a culturally situated Recovery Support Model that foregrounds the interconnected roles of personal agency, family involvement, professional engagement, and community support as drivers of recovery-oriented practice within the Thai mental health system.

## Introduction

Since the 1980s, mental health recovery has become a cornerstone of modern mental health policy internationally [1,2]. Three distinct types of recovery are recognised: clinical recovery, functional recovery, and personal recovery. Clinical recovery focuses on symptom remission [3,4]. Functional recovery encompasses vocational functioning, social participation, independent living, and housing stability, reflecting an individual’s capacity to engage meaningfully in everyday life beyond symptom remission [5]. In contrast, personal recovery is an ongoing process that emphasises individuals’ strengths and active involvement in their care. It encourages shared decision making, personal goal setting, and the pursuit of meaningful outcomes beyond the limitations of mental illness [6,7]. As a process of healing and transformation, personal recovery empowers individuals with mental health difficulties to live meaningful lives and reach their full potential within their chosen communities [8]. In line with the principles of deinstitutionalisation, supporting quality of life involves enabling people with mental health conditions to live in the ‘community’ rather than in an ‘institution’ [9].

Promoting personal recovery, hereafter ‘recovery’, in community mental health care requires robust support from community-based mental health services. Achieving meaningful recovery outcomes involves shifting from a predominantly biomedical model to a more holistic, recovery-oriented approach that prioritises individual strengths and overall well-being [10]. The success of such services relies on healthcare providers’ understanding of and attitudes toward recovery, as they play a central role in delivering care in close collaboration with service users [11,12]. A clear and shared understanding of the recovery concept is essential, as it informs and shapes mental health care practices. Although the concept of recovery has been examined in several Asian contexts, its interpretation remains highly diverse. Research on personal recovery among people experiencing mental illness in the region is still in its infancy. Further investigation is therefore required to elucidate how recovery is conceptualised and operationalised [13].

In Thailand, consensus on the definition and meaning of personal recovery remains limited. Further work is therefore warranted to validate the concept within the Thai context and to explore its culturally specific nuances and meanings. Moreover, little research has examined how key stakeholders, including service users, carers, and healthcare professionals, understand and experience recovery. Advancing mental health practice in Thailand towards a more recovery-oriented paradigm will require service users, practitioners, and researchers to cultivate a shared understanding of what effective recovery entails within their sociocultural context.

### Background

Between 2015 and 2023, mental health facilities across Thailand recorded 13,793,884 visits, with anxiety disorders, schizophrenia, and depression representing the three most common conditions among service users [14]. Thailand’s mental health system is structured around 13 regional mental health centres operating under the Department of Mental Health, within the Ministry of Public Health [15]. These centres play a pivotal role in coordinating and supporting community mental health services through collaboration with local healthcare networks, including provincial, district, and subdistrict hospitals [16,17]. At the primary care level, each subdistrict hospital typically employs one to two general nurses, one to two public health officers, and, in some cases, dental personnel and other support staff [18]. Each subdistrict hospital is responsible for the healthcare of 10–15 villages, depending on the district’s size and population density [18].

In Thailand, the community mental healthcare system is integrated within the broader public health system [19], creating a need for shared responsibility among providers of both physical and mental health services. As a result, mental healthcare in primary and community settings is often delivered by non-mental health specialist staff, such as general registered nurses and public health officers [18]. Thai general nurses and public health officers play a pivotal role in disease prevention and control at the frontlines of primary care [20]. In addition to these responsibilities, they work in close collaboration with mental health nurses from the district hospitals to support the provision of mental healthcare services within community settings.

To validate the concept of personal recovery within Thai community mental health care, it is essential to consider the perspectives of non-mental health professionals. Their insights help to illuminate the practical realities of delivering mental health services at the primary care level. At present, there are gaps in our understanding of recovery from the viewpoints of key service providers such as mental health nurses [21] and village health volunteers [22]. This study examined the concept of recovery from the perspectives of both mental health professionals, general healthcare professionals, as well as mental health service users and their carers. By drawing on real-world experiences, this study aimed to develop and culturally validate a shared, contextually grounded understanding of recovery within the Thai mental health system.

### Aim

To explore the thoughts, perceptions, and experiences of mental health service users, their carers, and healthcare professionals regarding mental health recovery and recovery support in Thailand.

## Methods

### Design

This study formed part of a larger project [23] that employed the Experience-Based Co-Design (EBCD) approach [24], which integrates participatory action research and user experience design, to co-design a culturally adapted recovery-oriented training intervention to improve recovery outcomes for mental health service users. The EBCD process consisted of two main parts encompassing six stages. Part 1 focused on gathering experiences (Stage 1: setting up, Stage 2: engaging staff and gathering experiences, Stage 3: engaging patients and gathering experiences), whereas Part 2 involved the co-design phase (Stage 4: co-design meeting, Stage 5: small co-design teams, Stage 6: celebration event) [25].

This paper presents Part 1 of the EBCD process, the experience-gathering phase which used semi-structured interviews and feedback workshops to explore mental health recovery and recovery support needs from the perspectives of service users and healthcare professionals. These findings informed the co-design of a recovery-oriented training intervention in Part 2 of EBCD process [23], with the aim of enhancing the quality of recovery-oriented mental health services. This paper is reported in accordance with the consolidated criteria for reporting qualitative studies (COREQ) guidelines [26].

### Study setting and recruitment

The study was conducted in Chiang Mai, Thailand, which is a major regional hub for healthcare, education, and culture, encompassing both urban and rural settings. Its diverse population is served by a comprehensive mental healthcare network, including psychiatric, provincial, district, and subdistrict hospitals. This context provided an ideal setting to examine the opportunities and challenges of implementing recovery-oriented practices within Thailand’s evolving mental healthcare system.

A purposive sample of service users, carers, and healthcare professionals were selected based on predetermined characteristics. Inclusion criteria for service users comprised individuals aged 18 and over with a diagnosis of mental illness or a substance-related disorder. Individuals experiencing an illness relapse at the time of recruitment were excluded. Carers were identified as individuals who cared for someone with a diagnosis of mental illness or a substance-related disorder. All healthcare professionals with experience of working with mental health service users in community mental health care settings were eligible to participate. Participants were recruited by the first author (NI), who had no prior connection to the study site and no preexisting relationship with any participant. Recruitment was conducted through face-to-face meetings during routine appointments at a mental health clinic in a district hospital between April 2025 and June 2025. Posters displayed within the clinic informed potential participants about the study as they were placed within the clinic setting, they were accessible only to individuals with experience of attending mental health services or their carers.

Consistent with Braun and Clarke’s reflexive thematic analysis, which prioritises depth of meaning and richness of interpretation over data completeness, formal data saturation was not sought [27,28]. Recruitment continued until sufficient depth and diversity of experience had been captured to meaningfully address the research objectives.

### Patient and public involvement

To strengthen the research design and methods, a Patient and Public Involvement (PPI) team was engaged to refine the data collection tools. Six PPI members were recruited through the first author’s (NI) connections with Thai mental health staff at a recovery centre in a psychiatric hospital in Thailand, as well as researchers in the field of mental health recovery. The team comprised a mental health nurse, a psychiatrist, a psychologist, a peer support specialist/researcher, a person with lived experience of mental illness, and a carer. Their involvement ensured that the data collection tools were clear, relevant, and linguistically appropriate, while also helping to minimise potential stigma arising from cultural and linguistic differences during the interviews.

### Data collection

The first author (NI), trained in EBCD and qualitative interview techniques, conducted all 30 interviews, each lasting approximately one hour. Interview topic guides (**see** S1 File) were initially developed in English by the research team and informed by the CHIME mental health recovery framework [29], the Global INSPIRE measure [30], the Brief INSPIRE questionnaire [31], the Recovery Self-Assessment measure [32], and findings from a review of factors influencing the delivery of recovery-oriented practice [12]. The interview guides were translated into Thai by NI with input from the PPI team, then piloted and refined with six PPI members to ensure clarity, cultural appropriateness, and sensitivity.

Prior to the interviews, participants with mental health condition completed a version of the Global INSPIRE questionnaire [33] which had been translated into Thai for the purposes of this study to provide insight into participants’ experiences of mental health recovery. This measure was initially translated into Thai by NI, with the back-translation verified by a member of the original development team at the University of Nottingham. The translation was further refined based on feedback from six PPI members. As the Global INSPIRE Thai version had not been formally validated, the measure was used solely as a discussion prompt to explore personal recovery among participants who might be unfamiliar with the concept. It provided a structured framework to guide conversations about personal recovery, rather than generating generalisable findings.

Semi-structured interviews were conducted by the first author (NI) in formal Thai or the Northern Thai dialect spoken in Chiang Mai, according to participants’ preferences and took place face-to-face at the hospital, in participants’ homes, or via telephone. All sessions were audio recorded with permission. Core questions explored participants’ perceptions and experiences of mental health recovery and recovery support, in alignment with the primary research objectives. With consent, five service users and three carers were additionally video recorded as part of the broader EBCD study to produce a catalyst film for use in co-design workshops [24].

Following the interviews, all participants were invited to take part in feedback events, a core component of the EBCD experience-gathering phase. These events provided an opportunity for participants to confirm whether the experiences identified in earlier stages accurately reflected their perspectives, thereby enhancing the credibility and trustworthiness of the data interpretation. During the session, preliminary findings from the interviews were presented for participants’ review, comments, and suggestions. Participants were also encouraged to share any additional insights or highlight new ideas that had not emerged during the interviews.

### Data analysis

All interviews were transcribed verbatim and translated from Thai and Northern Thai into English by a professional transcriber and translator who was fluent in formal Thai, the Northern Thai dialect, and English. Transcription was treated as a constructive rather than mechanical process, recognising that selectivity and interpretation inevitably shape the conversion of spoken data into text [34,35]. To minimise potential errors and bias, the first author (NI) met with the transcriber prior to commencement and maintained regular communication throughout, ensuring familiarity with local expressions, cultural meanings, emotional nuances, and technical terminology. NI reviewed all transcripts to verify accuracy, contextual fidelity, and to address any cultural or linguistic ambiguities through discussion with the transcriber and the research team (NI, AG, ML).

Translation in qualitative research requires not only linguistic conversion but preservation of context, intention, and cultural meaning [36,37]. NI met with the translator prior to commencement and maintained regular communication throughout to ensure familiarity with the research context and participants’ cultural background. A glossary of study-specific terminology including terms such as mental health recovery and personal recovery, and culturally specific relational terms was provided to support translation accuracy. NI reviewed and verified all English transcripts against the original Thai to ensure accuracy, consistency, and conceptual equivalence.

The first author (NI) conducted an independent inductive analysis using Braun and Clarke’s six-phase reflexive thematic analysis comprising: familiarisation with data, coding, generating initial themes, reviewing themes, defining and naming themes, and producing the final report [38,39]. During familiarisation, NI listened to the audio recordings and read all transcripts in both Thai and English to develop a thorough understanding of the data. In the coding stage, English transcripts were used to enable the wider research team (AG, ML) to engage with the data. However, both language versions were considered throughout to support nuanced interpretation and minimise language bias [40,41]. NI conducted a line-by-line analysis of all transcripts using NVivo software (version 15). Initial codes were organised according to the two research objectives: participants’ experiences of recovery and recovery support, facilitating identification of similarities and differences across the dataset. Consistent with EBCD methodology, touchpoints were also identified. Touchpoints refer to emotionally significant moments within participants’ narratives that illuminated aspects of care perceived as meaningful, impactful, or requiring improvement [24,42]. NI generated initial themes and subthemes by consolidating related codes and touchpoints, which were subsequently reviewed and refined through regular meetings with the research team (AG, ML). Themes were iteratively revised until consensus was reached, and findings were synthesised and illustrated with participant quotes.

### Ethical considerations

Ethical approval for the study was granted by King’s College London (Reference number: HR/DP-24/25–45539) and permission for data collection was obtained from Chiang Mai Provincial Public Health Office (CM0033.010/2721). Data collection was conducted between April 2025 and September 2025. All participants received an information sheet outlining the study’s purpose, procedures, and confidentiality measures, and written informed consent was obtained prior to participation in both the interviews and feedback workshops.

### Rigor and reflexivity

To ensure the rigour and trustworthiness of the study, crystallisation was employed by drawing on multiple data sources, researchers, and analytical perspectives to broaden inquiry and deepen interpretation [42]. Multiple data sources were achieved through the inclusion of diverse participant perspectives from service users, carers, and multidisciplinary healthcare professionals, alongside the use of individual interviews and feedback workshops. The two feedback workshops, served as participant reflections [42], providing participants with opportunities to critique, affirm, and refine the findings in support of collaborative interpretation. Researcher diversity was achieved through a multidisciplinary research team with diverse academic and professional backgrounds. NI is a Thai male academic trained in mental health and general nursing with a research interest in mental health recovery. AG and ML are Irish and English female academics with expertise in qualitative research and mental health recovery.

Throughout the data collection, we acknowledged that NI’s professional background as a Thai and UK trained mental health nurse may have shaped interview dynamics, potentially influencing participant openness and how participants positioned themselves in relation to clinical authority. To minimise this influence, NI established rapport, adopted a non-judgemental and open-ended interviewing style, highlighted the value of participants’ lived experience and professional expertise, and emphasised his role as an independent researcher. NI maintained a reflexive stance throughout, engaging in continuous self-reflection and critical dialogue with the research team.

## Results

### Characteristics of participants

In total, 30 participants were interviewed comprising five general nurses, one mental health nurse, two psychologists, seven public health officers, five carers, and ten mental health service users. In addition, 11 participants (eight healthcare professionals and three service users) participated in the feedback workshops. The interviews lasted between 23 and 70 minutes. Participants’ characteristics and demographic information are presented in Table 1.

**Table 1:** Participant characteristics and demographic information.

Characteristic	HCPs (n = 15)	PwM (n = 10)	Carers (n = 5)
Age, mean (range)	34.9 (25-50)	56.0 (37-72)	67.6 (60-75)
Male, n (%)	6 (40)	3 (30)	2 (40)
Female, n (%)	9 (60)	7 (70)	3 (60)
Thai ethnicity, n (%)	15 (100)	10 (100)	5 (100)
Buddhist, n (%)	13 (86.7)	9 (90)	5 (100)
Undergraduate or postgraduate degree, n (%)	14 (93.3)	2 (20)	0 (0)
Time in mental health/community care, mean (range)	6.4 (0.5-28)	7.5 (1.5-23)	13.6 (5-23)*
**Healthcare professionals only**			
**Characteristic**			**HCPs (n = 15)**
Work in district hospital			4 (26.7)
Work in sub-district health promoting hospitals			11 (73.3)
General nurses			6 (33.3)
Mental health nurse			1 (6.7)
Psychologists			2 (13.3)
Public health officers			7 (46.7)

**Note:** HCP = healthcare professionals; PwM = people with mental illness (mental health service users).

*For carers, this refers to time caring for PwM.

Four overarching themes were developed from the perspectives of mental health service users, carers, and healthcare professionals, each supported by corresponding subthemes that further illustrate the data. A summary of emergent themes, subthemes, codes, and touchpoints is presented in Table 2. Additional and supporting quotes from participants are presented in S2 File.

**Table 2:** Coding framework of touchpoints, themes, and subthemes of service users and healthcare professionals.

Themes	Subthemes	Codes and touchpoints	Subthemes	Codes and touchpoints
**Theme 1: Conceptions of mental health recovery**	***Service users***		***Healthcare professionals***	
	**SU1.1:** Recovery begins with the hope of being free from clinical symptoms	Taking medication helps them to get better	**HCP1.1:** Clinical recovery must come first	The patient must go through the proper treatment process first
		Recovery means a reduction in relapses or readmissions		The patient’s psychiatric symptoms must be stabilised first
		Living without mental health conditions	**HCP1.2:** There is a strong alignment between functional and personal recovery	People in recovery can often resume basic self-care tasks like bathing, doing laundry, and working
		Hope that advances in medicine and technology will improve treatment and reduce clinical symptoms		Recovery would be when patients can live in the community, can work to support themselves, and are not causing problems for others
	***Joint subthemes***			
	**JST1.1:** Personal recovery could mean…	‘Being happy with one’s own mental health’	**JST1.1:** Personal recovery could mean…	‘A sense of control we have over our life’
		‘Having good quality of life, better health, better life’		‘Being able to live independently’
		‘Being able to control my life’		‘Having strength within our mind’
		Being adaptable to the surrounding environment and society		‘Living confidently’
		Being able to connect and interact with other people		‘Living with illness’
		Having hope		‘Not causing problems for others’
		Living independently		‘Not feeling lonely’
		Living life without bothering or causing trouble to others		Being adaptable and resilient
				Having a good mindset
				Having life goal/s
				Living with hope
				Maintaining balance
				Reintegrate into society or community
				Responsible for themselves and others
				Return to live a normal and peaceful life
**Theme 2: Attitudes towards mental health recovery**	***Service users***		***Healthcare professionals***	
	**SU2.1:** Staff are regarded with deference and authority	‘Whatever the doctor orders, we do’	**HCP2.1:** Working in mental health care is challenging	Fear of working in psychiatric and mental health care
		Respect staff and follow their advice		‘It’s a stressful work’
				‘Working with substance misuse is risky’
				Not confident to provide mental health care
				Thai people have bias towards people with mental illness
			**HCP2.2:** Multi-sectoral collaboration and shared responsibility for mental health care	More awareness and involvement from organisations beyond just public health
				‘Everyone sees it as a shared responsibility to help patients access treatment as early as possible’
			**HCP2.3:** Belief in the possibility of recovery for all	‘Regardless of which group of psychiatric patients they belong to, everyone has the chance to recover’
				It is not just about medication: If someone stops taking medication, they can still recover
	***Joint subthemes***			
	**JST2.1:** Mental health receives limited attention and prioritisation	Mental health receives less focus than physical health	**JST2.1:** Mental health receives limited attention and prioritisation	Subdistrict hospital does not emphasise mental health
		‘Mental health still isn’t seen as serious enough’		Community mental health service is underdeveloped
				Mental health work has less weight than physical health
**Theme 3: Characteristics of a successful recovery journey**	***Service users***		***Healthcare professionals***	
	**SU3.1:** ‘It’s more about how we carry ourselves’	‘It depends on how we behave’	**HCP3.1:** ‘It has to start with patients themselves’	Accepting their conditions or illness
		Being optimistic		Having self-awareness
		Being satisfied with life		Willing to act or change behaviour
		Just let go with things in life		Being able to have their own choice
		‘Treat illness as it comes as part of everyday existence’		Participating with others
		Acceptance of illness is a good starting point	**HCP3.2:** Recovery requires collective community involvement	If community understand and doesn’t stigmatise patients, it can help support patient’s recovery
	**SU3.2:** Feeling loved and supported	Feeling loved and supported by people around me		Accepting patients and give a chance for them to live in the community
	**SU3.3:** Preserving self-worth and contributing to others	Being financially secure and less dependent		Local shops help by not selling alcohol to patients
		Having a job and savings brings a sense of security		Village health volunteers support patients’ recovery in the community
		Being able to provide support to others		Village leaders support recovery
			**HCP3.3:** Staff must possess recovery-oriented knowledge, attitudes, competencies and skills	‘Giving encouragement’
	**SU3.4:** Staying connected with people and nature	Connecting and interacting with other people		We should be careful about our words
		Connecting with nature		Helping people (with substance misuse) to set goals
	**SU3.5:** Feeling accepted and treated like everyone else	‘Don’t forbid us from doing everything’		Staff compassionate to help
		‘Don’t treat us differently from anyone else’		Having positive attitudes to support recovery
				Staff must be knowledgeable in (recovery-oriented) mental health care
	**SU3.6:** Reducing or stopping alcohol consumption	Recovery could start from ‘stop drinking’		Staff must be skilful
		Family has to control alcohol consumption		Allowing patients and carers to get involved in their care
	**SU3.7:** Religious support as a source of recovery	It brings mindfulness		Using role model
		The karma that struck me eased, and things got better		Staff should be ‘neutral’
		Some people abstain from alcohol on Buddhist holy days		
	***Joint subthemes***			
	**JST3.1:** Family as a central factor in recovery	‘It has to be the people in the house’	**JST3.1:** Family as a central factor in recovery	There is no one else left in the family: If relatives do not take care of patients, then who will do it?
		Family act as moderator for patents and staff		Bringing patient to the appointment
		Having a life partner would provide good support and reduce loneliness		Helping them to have medication at home
		Openness to/from family		Encouragement from the closest people really matters
	**JST3.2:** Peer support seems promising	‘There should be peers to exchange experiences’	**JST3.2:** Peer support as a promising approach	If someone has recovered and can share their experience, it might help others improve
		‘If you’re not in this situation, you wouldn’t understand’		We (staff) haven’t been in their shoes and that we may not fully understand the patients the way someone with lived experience can
**Theme 4: Factors impeding recovery**	***Service users***		***Healthcare professionals***	
	**SU4.1:** Barriers that discourage recovery	Feeling embarrassment being in mental health service	**HCP4.1:** Patients’ lack of engagement in their own recovery	Not accepting their illness
		Feeling that medication sometimes makes daily activities harder		Experiencing self-neglect
		People with mental illness can’t live alone and need someone to support them		The patient is not in a condition to discuss recovery
		Recovery is difficult for older people		‘Refused to get treatment’
	**SU4.2:** Insufficient support for recovery	‘No one comes to guide us’		Lack willingness to stay longer for additional (recovery-oriented) care
		Lack health education support	**HCP4.2:** Family can’t provide care anymore	Family lack of readiness and willingness
		Subdistrict hospital lacks readiness to provide support		‘Patients being neglected’
	**SU4.3:** Mental health care remains distant from recovery-oriented practice	More actively recovery support from staff is needed	**HCP4.3:** Community reluctance to reintegrate individuals with mental illness	Patient being rejected
		Care should be more person centred		Patient usually causes recurrent troubles to others
		People with mental illness are rarely asked about their hopes or goals in their life		
	**SU4.4:** Inequality in healthcare service	Disability support is sometimes selective		
		Support provided is unfair	**HCP4.4:** Staff challenges in supporting patient recovery	‘Lack of staff’
	**SU4.5:** Challenges in addressing alcohol misuse	When it comes to alcohol ‘they just won’t listen at all’		‘Paperwork removes us from patients’
		All money is spent on alcohol		Staff in subdistrict hospitals lack of specialty in mental health
		Festivals increase the chance of alcohol consumption		There is no indicator for mental health recovery
				Broad and non-specific scope of duty of care
				Lack of implementation
				Lack of psychiatrist in district and subdistrict hospitals
				Over workload
				Time constraint
	***Joint subthemes***			
	**JST4.1:** Employment barriers for people with mental illness	‘I was very discouraged’ there weren’t jobs available for people with mental illness	**JST4.1:** Employment barriers for people with mental illness	Employer is reluctant to hire
				Psychiatric conditions can affect physical ability (work)
				Patient is unpredictable like ‘three days on, four days off’
	**JST4.2:** Peer influence on relapse among people with substance use difficulties	‘Friends have a lot of influence’	**JST4.2:** Peer influence on relapse among people with substance use difficulties	The old friend might influence and they go back to the same cycle
		Gangsters hinder recovery		The same context, the same community, the same society, then they would reverse to the same pattern

**Note:** SU = subtheme of service user (mental health service users and carers), HCP = subtheme of healthcare professionals, JST = joint subtheme

### Theme 1: Conceptions of mental health recoverya

#### SU1.1: Recovery begins with the hope of being free from clinical symptoms.

Service users and carers primarily understood recovery through a biomedical lens, equating improvement with the reduction or absence of clinical symptoms and emotional distress. Recovery was commonly associated with freedom from symptoms such as worry, depression, panic, suicidal ideation, and obsessive thinking, and was viewed as the necessary foundation for regaining happiness, stability, and a sense of normal daily life. Carers similarly gauged recovery through observable changes in mood, behaviour, and daily functioning, indicating that both subjective wellbeing and visible functional improvement shaped their understanding of progress.

#### HCP1.1: Clinical recovery must come first.

Healthcare professionals largely understood recovery as a process contingent on prior symptom stabilisation. Clinical stability was described in terms of behavioural control, the capacity to process and retain information, and the ability to communicate effectively with staff. Functional improvement and personal recovery were consequently positioned as sequential to rather than concurrent with symptom management, even from the point of crisis presentation.

*“During the acute or crisis phase, it’s really not manageable. It’s beyond our capacity at the subdistrict health promoting hospital. The patient must go through the proper treatment process (in psychiatric hospitals) first. Only then we can start having meaningful conversations.”*

*- HCP02*

#### HCP1.2: There is a strong alignment between functional and personal recovery.

Healthcare professionals conceptualised recovery not only as clinical improvement but also as the restoration of functional capacity. “Functional recovery” was consistently framed around independence in self-care, the ability to manage daily routines, and returning to employment for financial self-sufficiency and family provision. These accounts suggest that symptom reduction alone was not considered sufficient; recovery was evaluated through individuals’ capacity to resume socially expected roles and responsibilities. Recovery was therefore understood as the process of reintegration into valued social roles, rather than simply achieving clinical stability.

*“At the very least, they should be able to take care of their basic daily needs. If it goes better, they might even help with family tasks. What we hope for is that they can live independently. Then they can work, take care of themselves, start helping their family. Some can even return to normal functioning we’d call it full recovery.”*

*- HCP05*

#### JST1.1: Personal recovery could mean….

Personal recovery was described as closely associated with adaptability and resilience, enabling individuals with mental illness to feel content and maintain a sense of control over their mental health. Recovery was understood as being rooted in inner strength and the ability to manage one’s life despite ongoing mental health difficulties. It often began when individuals developed a more positive mindset, recognised the need for change, and set clear life goals with hope for the future. As one healthcare professional reflected, recovery started when a person realised, “I should be better”. Recovery did not mean the complete elimination of mental illness, but rather the ability to live independently, confidently, hopefully, and peacefully while continuing to manage ongoing symptoms and challenges. The ultimate goal of personal recovery was seen as successful reintegration into society and community life, where individuals could live meaningfully without creating recurrent difficulties for themselves or others.

**Fig 1:**
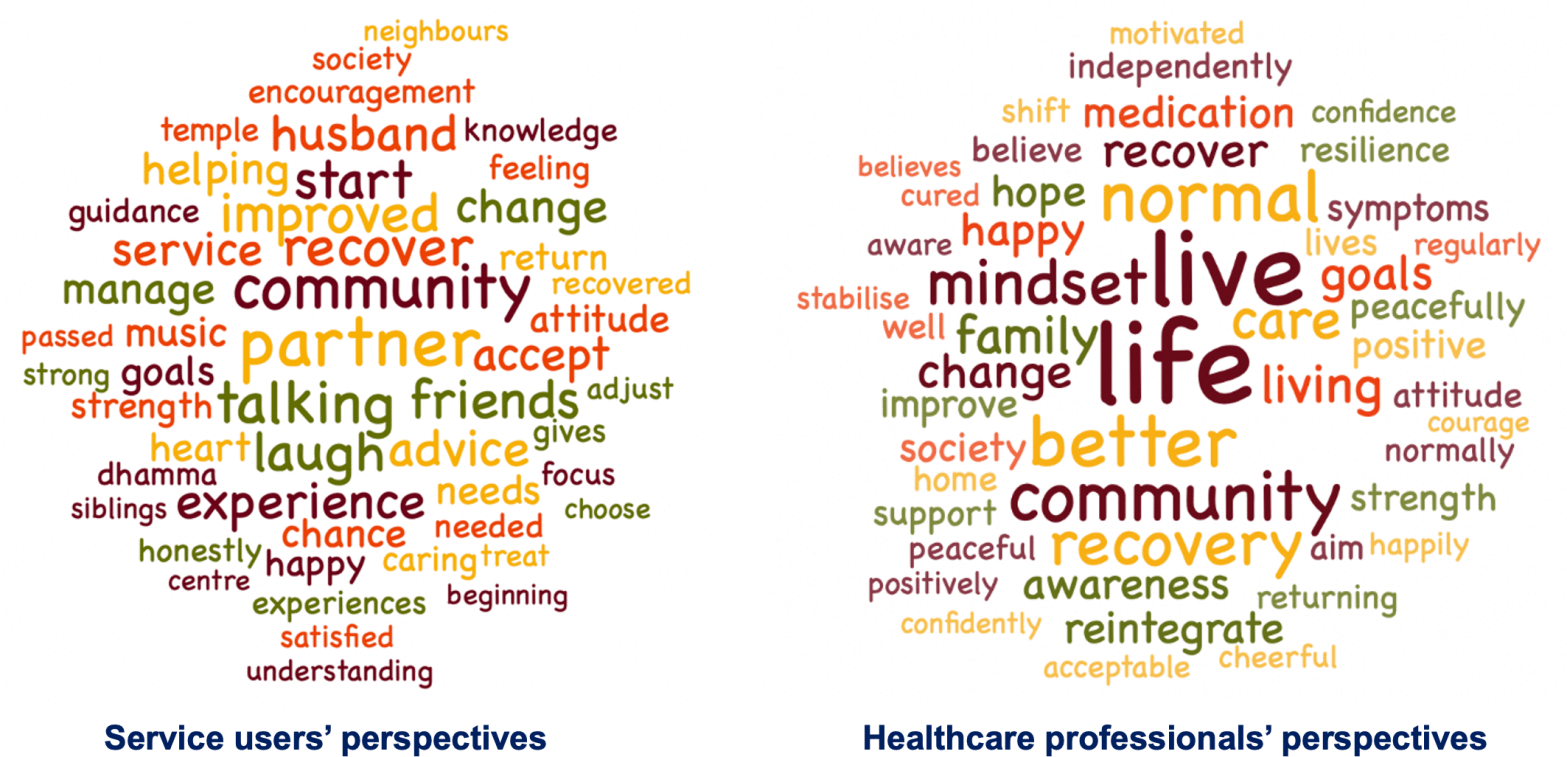
Frequently used words in discussions of recovery.

Fig 1 presents a word cloud illustrating the most frequently occurring terms used by participants when describing personal recovery. This is not intended as a standalone analytical tool but offers a visual overview of dominant recovery-related concepts that complements the thematic analysis and contextualises participants’ shared understanding of recovery.

### Theme 2: Attitudes towards mental health recovery

#### SU2.1: Staff are regarded with deference and authority.

Service users and carers consistently perceived healthcare professionals as the most legitimate source of knowledge and authority in mental health recovery. Doctors and healthcare staff were described as highly educated and competent, which fostered a strong tendency to trust professional judgement and follow treatment recommendations with limited questioning. Recovery was consequently positioned within a provider-led model, in which treatment compliance was understood as the primary route to improvement.

#### HCP2.1: Working in mental health care is challenging.

Healthcare professionals described workforce sustainability in community mental health care as constrained by limited specialist training and the emotional demands of practice. In Thailand, mental health nursing specialisation occurs only post-registration and recruitment remained difficult as many general nurses were reluctant to transition into psychiatric settings, largely due to fear, stigma, and uncertainty about managing the psychological demands of this work. Despite these challenges, compassion and a strong sense of responsibility towards mental health service users were identified as key factors sustaining staff engagement, suggesting that workforce participation was often driven more by personal commitment as much as formal preparation.

Participants also highlighted the complexity of supporting service users with substance misuse-related problems, which were perceived as particularly high risk. These cases intensified concerns about staff safety and heightened anxiety around community care delivery, particularly where there were fears of aggression or unpredictable behaviour.

*“The main challenge is all about psychiatric patients here (in the community) who have issues with substance misuse. That makes our work risky and dangerous in providing care, because we don’t know what if the patient recovers and returns (from jail or substance rehabilitation hospital), they might harm (us) or what if they still hold resentment or anger toward us?”*

*- HCP01*

#### HCP2.2: Multi-sectoral collaboration and shared responsibility for mental health care.

Healthcare professionals viewed mental health recovery as a shared responsibility requiring coordinated involvement from government agencies, community organisations, families, and other public services. Increasing awareness of the wider social determinants of mental health was seen as strengthening this collective approach. Initiatives such as the Serious Mental Illness (SMI) pathway were described as examples of effective multi-sectoral collaboration, enabling individuals experiencing acute psychiatric symptoms to be referred and admitted promptly without legal barriers. This was perceived as improving rapid access to emergency psychiatric care and strengthening inter-agency cooperation in crisis management.

#### HCP2.3: Belief in the possibility of recovery for all.

Healthcare professionals consistently emphasised that recovery was possible across all mental health conditions. While some staff considered recovery from substance misuse more challenging than from mood disorders such as depression, diagnostic category alone was not seen as determining recovery outcomes. Instead, they emphasised the importance of surrounding support systems, particularly family involvement, continuity of care, treatment adherence, and access to ongoing support. Recovery was therefore understood as contingent on enabling environments rather than illness severity.

*“Regardless of which group of psychiatric patients they belong to, everyone has the chance to recover. It depends on the supportive factors. It is not just about medication. If someone stops taking meds, they can still recover. Or if someone has schizophrenia, even with the illness, if their family provides proper care and they take their medication and attend appointments, then all groups have the same chance.”*

*- HCP11*

#### JST2.1: Mental health receives limited attention and prioritisation.

Participants described mental health as receiving lower institutional priority than physical health conditions, within the Thai healthcare system, particularly in subdistrict hospitals where community mental health services remained limited and underdeveloped. In contrast to areas such as non-communicable disease management, maternal and child health, vaccination programmes, and elderly care, mental health lacked clear performance indicators and formal evaluation mechanisms, reducing its visibility and organisational priority. Mental health responsibilities were consequently often assigned to general nurses without specialist psychiatric training, reinforcing the marginal status of mental health within routine service delivery.

*“In my opinion, (community) mental health services are still quite limited and underdeveloped. As I mentioned, many people experience both physical and psychological symptoms, but most people including health personnel tend to focus only on physical health. […] Right now, (community) mental health services are still minimal and not comprehensive.”*

*- HCP11*

### Theme 3: Characteristics of a successful recovery journey

#### SU3.1: ‘It’s more about how we carry ourselves’.

Service users frequently positioned recovery as shaped by personal attitudes and self-determination rather than clinical treatment alone. Acceptance of mental illness was described as an essential first step, involving recognition of the condition as part of life and learning to live alongside it rather than allowing it to define one’s identity. Recovery was associated with sustaining optimism and developing the capacity to move beyond distress rather than remaining fixed in the role of a patient.

*“When we’re in society, it depends on how we behave. It’s not like, just because they say we have schizophrenia, we act all crazy and live without care. If we do that, we won’t be able to be part of society, right? I didn’t think that way. I don’t carry it with me. I think of myself as normal, just like anyone else. […] Just because the doctor said I had a mental illness doesn’t mean I have to act like a patient. Just be normal, like before I got sick.”*

- PwM03

#### SU3.2: Feeling loved and supported.

Service users described love, encouragement, and trust from significant others as central relational conditions supporting recovery. Supportive family relationships characterised by care, respect, and emotional reassurance fostered a sense of belonging, safety, and personal value, which were seen as important for sustaining hope and gradual improvement in wellbeing.

Family harmony was particularly valued as a source of emotional stability, with the absence of conflict, criticism, and tension, especially within extended family relationships was considered essential for maintaining positive mental health.

#### SU3.3: Preserving self-worth and contributing to others.

Service users positioned financial stability and independence as important markers of recovery, linking the ability to work, earn an income, manage daily expenses, and repay debts with a sense of security, dignity, and reduced psychological distress. Economic independence was associated not only with practical stability but with self-worth, and the restoration of control over everyday life.

Recovery was also closely connected to the capacity to contribute to others and remain socially engaged. Helping others, participating in community activities, and undertaking voluntary or public work were described as meaningful sources of purpose and fulfilment, strengthened wellbeing by enabling individuals to give as well as receive support.

*“Really, if you help others and do it with sincerity, anyone who has the heart to help others will feel happiness from it. It brings joy. It depends on the person and your intention too. If you genuinely want to help others, when you get to do it, it feels like you fulfilled that purpose […].”*

*- PwM09*

#### SU3.4: Staying connected with people and nature.

Service users described recovery as supported by both social engagement and connection with nature. Regular interaction with others was viewed as important for maintaining a sense of normality, reducing preoccupation with illness, and preventing social isolation. Social participation was seen to relieve negative thinking, provide emotional distraction, and protect against distress, including suicidal ideation. Connection with nature was similarly described as a source of restoration and emotional balance. Activities such as caring for animals, walking in forests, and gathering herbs or mushrooms helped participants feel calm, grounded, and mentally refreshed. Recovery was therefore supported not only through interpersonal relationships but also through everyday engagement with natural environments that provided comfort, purpose, and psychological relief.

#### SU3.5: Feeling accepted and treated like everyone else.

Service users linked recovery to the preservation of autonomy and the ability to maintain an ordinary social identity beyond mental illness. Recovery was strengthened when individuals could manage daily activities independently, make their own decisions, and participate in everyday life without constant supervision or overprotection. Being able to go out, complete personal errands, and live without excessive monitoring reinforced feelings of competence and self-efficacy.

Equally important was how others responded to them. When family members and those close to them avoided treating them as different, fragile, or permanently defined by diagnosis, service users felt more respected, valued, and socially included.

*“[…] because the people close to me didn’t treat me differently or specially. They treated me the same as before, nothing really changed. I could still go out, do my own errands, and live my life without needing anyone to constantly look after me. It wasn’t as if I had to depend on anyone or be supervised all the time.”*

*- PwM03*

#### SU3.6: Reducing or stopping alcohol consumption.

For some participants, recovery was strongly associated with reducing or stopping alcohol use, particularly where alcohol was perceived to undermine daily functioning, emotional stability, and the ability to manage everyday life. Family support was identified as central to this process, with relatives playing an important role in encouraging abstinence, providing practical monitoring, and offering emotional guidance. Recovery from alcohol-related difficulties was therefore understood as both an individual and relational process, in which sustained improvement depended heavily on close family involvement.

*“If he (patient) could just stop drinking, that’s the only thing, he would probably get better. […] The biggest worry is alcohol. If he quits, he can manage his own life. If alcohol is out of the picture, then I’m not worried anymore.”*

*- Carer01*

#### SU3.7: Religious support as a source of recovery.

Spiritual and religious practices were identified as important supports for personal recovery, fostering mindfulness, acceptance, and a sense of purpose. Activities such as participating in rituals, listening to Buddhist teachings, chanting, and dedicating merit were described as promoting reflection, emotional regulation, and forgiveness, while helping individuals cope with past negative experiences or the consequences of karma. Religious practices were also linked to structure and behavioural self-regulation. Routine observances, such as abstaining from alcohol on holy days, were seen to strengthen self-discipline and support gradual positive change in everyday life.

*“When I go to temples, pay respects to the Buddha, light incense, and make a vow in front of the main Buddha statue, then pour water to dedicate merit on holy days, I feel fulfilled. I feel content because I’ve done good deeds. The bad karma that affected me seems to ease and lessen. […] Once I felt that the merit I made helped me, it softened things. The karma that struck me eased up. Things got better.”*

*-PwM04*

#### HCP3.1: ‘It has to start with patients themselves’.

Healthcare professionals positioned recovery as beginning with the individual’s recognition and acceptance of their mental health condition. Acknowledging illness was seen as a necessary first step that enabled engagement with counselling, medication, and other forms of support, allowing individuals to regain insight and develop a more hopeful and engaged orientation towards recovery. Without this initial acceptance, healthcare professionals felt that meaningful therapeutic progress was difficult to achieve.

However, willingness alone was not considered sufficient. Recovery was understood to require active engagement in treatment and recovery-oriented behaviours, rather than a passive acceptance of care. This reflects a view of recovery that prioritised personal responsibility and behavioural participation, in which improvement was closely linked to the individual’s readiness to change and openness to receiving support.

*“I think it has to do with openness to accept help. Like I said, it depends on whether the patient wants to get better. If they want to be normal, it’s about whether they’re ready to open up. But even for psychiatric patients, if they open up, they could also recover.”*

*- HCP04*

#### HCP3.2: Recovery requires collective community involvement.

Successful community reintegration was understood as requiring collaboration beyond formal healthcare services, particularly through partnerships with local leaders, village health volunteers, and the wider community. Village heads, subdistrict chiefs, and village health volunteers were described as key actors in monitoring wellbeing after discharge, providing updates on service users’ conditions and helping to coordinate responses when difficulties arose. This was especially important in community settings where limited staffing reduced the capacity for continuous professional follow-up.

For individuals experiencing alcohol misuse, restricting community access to alcohol through engagement with local retailers was also viewed as an important relapse prevention strategy. More broadly, participants emphasised that openness, understanding, and non-stigmatising attitudes within the community were essential for helping mental health service users feel accepted and supported.

#### HCP3.3: Staff must possess recovery-oriented knowledge, attitudes, competencies and skills.

Healthcare professionals described recovery-oriented care as dependent not only on clinical knowledge but also on relational and communication skills that supported trust, dignity, and engagement. Careful use of language and attention to recovery-oriented terminology were seen as important for reducing stigma and fostering respectful interactions with patients and families. Kindness, compassion, unconditional acceptance, and sustained belief in the person’s capacity for recovery were consistently identified as central to effective practice.

These interpersonal approaches were understood to strengthen therapeutic relationships and encourage greater participation from service users and carers in treatment discussions and future planning. In terms of structured goal setting, this was most commonly embedded within care pathways for people with alcohol dependence. However, some healthcare professionals described applying similar approaches more broadly, supporting individuals in identifying personal goals related to employment, daily functioning, and treatment engagement. This suggests that recovery-oriented care was understood as encompassing both supportive therapeutic relationships and practical collaboration around meaningful life goals.

#### JST3.1: Family as a central factor in recovery.

Participants consistently positioned family involvement as central to successful recovery. As the people in most continuous contact with the individual, family members were seen as essential in providing encouragement, emotional support, practical guidance, and ongoing monitoring of wellbeing. Effective family support depended on relatives having an understanding of the condition, maintaining open communication, and remaining actively engaged even during periods of frustration or exhaustion. Patients whose families actively mediated between them and their healthcare professionals were perceived to have stronger treatment engagement and better recovery outcomes than those living alone.

#### JST3.2: Peer support as a promising approach.

Participants identified peer support as a valuable component of recovery, particularly because individuals with lived experience could offer forms of understanding and guidance that professionals alone could not provide. Seeing someone who had experienced mental illness and progressed in their own recovery helped patients develop hope and recognise that improvement was possible. Peer supporters were also seen as able to provide practical advice on managing daily life, communicating with family, and navigating challenges that were often difficult to address through clinical support alone. Peer relationships helped normalise mental health difficulties, reduced feelings of isolation, and provided reassurance that individuals were not facing recovery alone. Healthcare professionals recognised that experiential knowledge offered a distinct form of credibility, particularly in fostering hope and helping patients feel genuinely understood.

*“[…] Because sometimes, we ourselves haven’t been in their shoes. Sometimes we may not fully understand the patients the way someone with lived experience can, as their peer.”*

*- HCP05*

### Theme 4: Factors impeding recovery

#### SU4.1: Barriers that discourage recovery.

Service users identified multiple barriers to sustained recovery, encompassing both social and practical challenges. Stigma and embarrassment were described as significant factors delaying help-seeking, with some individuals avoiding mental health clinics due to fear of being seen, judged, or labelled by others. This suggests that concerns about social perception continued to restrict timely access to support.

Medication management also presented difficulties, particularly when psychiatric treatments caused excessive drowsiness that interfered with work, daily functioning, and independence, sometimes necessitating adjustments to balance symptom control with quality of life. Recovery was perceived as especially challenging for older adults, where age-related cognitive decline and the emergence of new symptoms complicated both treatment and self-care. Individuals living alone were additionally seen as more vulnerable to poor medication adherence and declining wellbeing due to the absence of family monitoring and everyday support. Together, these accounts illustrate how recovery was shaped not only by treatment but also by stigma, ageing, and the availability of practical and relational support.

#### SU4.2: Insufficient support for recovery.

Recovery was described as more difficult when carers received limited guidance from external services, leaving families to manage much of the caregiving responsibility alone. Relatives were often expected to monitor symptoms, supervise medication, and respond to complications such as alcohol-related poor nutrition, while relying on trial and error rather than clear professional guidance. This created significant uncertainty, emotional strain, and feelings of isolation, particularly when carers were unsure where to seek help and community services offered limited proactive outreach.

Participants also highlighted structural barriers within subdistrict hospitals, including limited medication availability, insufficient resources, and high patient volume, which undermined continuity of care and access to timely support. In some cases, referral to district hospitals created additional travel demands and financial pressure for both patients and families.

#### SU4.3: Mental health care remains distant from recovery-oriented practice.

Participants described recovery support within mental health services as predominantly centred on medication management, with limited ongoing dialogue, education, or collaborative guidance. Interactions with healthcare staff were often brief and focused primarily on prescription adjustments, while the structured self-management support commonly available for other long-term conditions, such as diabetes education was largely absent. As a result, patients and carers were frequently left to seek information independently and manage recovery with minimal professional support.

#### SU4.4: Inequality in healthcare service.

Carers described significant frustration with the perceived inconsistency and selectivity of disability and social welfare support, which often left families carrying the full burden of long-term caregiving. Many felt that formal support systems were unreliable or inaccessible, despite the substantial emotional, physical, and financial demands of caring for a person with mental illness. This reinforced a sense that recovery support depended on family capacity rather than equitable access to external assistance.

Some carers reported being advised by healthcare staff to simply continue providing care indefinitely, which intensified feelings of neglect, helplessness, and abandonment. Family members also perceived inequalities in how support was distributed, with those connected to health workers perceived as more likely to receive assistance, while their own evident needs were unmet. These experiences suggest that perceived institutional unfairness further undermined carers’ ability to sustain recovery within the family environment.

#### SU4.5: Challenges in addressing alcohol misuse.

Carers described alcohol misuse as one of the most persistent barriers to recovery, particularly when repeated advice, medical recommendations, and family efforts failed to produce behavioural change. Resistance to reducing alcohol use generated considerable frustration and emotional strain, with carers reporting that individuals often failed to recognise the impact of their drinking on daily functioning and recovery progress.

Financial management was a significant concern, as any money provided was frequently spent on alcohol, undermining household stability and treatment continuity. Social and cultural occasions including New Year, Songkran, and funerals were identified as high-risk situations where alcohol consumption was normalised and difficult to avoid, often precipitating relapse. These accounts highlight how recovery from alcohol-related difficulties was shaped not only by individual behaviour but also by broader social and cultural environments that normalised and sustained drinking practices.

#### HCP4.1: Patients’ lack of engagement in their own recovery.

Healthcare professionals frequently identified limited illness recognition and treatment engagement as major barriers to recovery. Many patients were described as not accepting their mental health condition, often leading to medication refusal, missed appointments, and disengagement from ongoing care, making recovery more difficult to sustain.

Among people experiencing substance misuse, these challenges were perceived as particularly pronounced. Healthcare professionals described how some individuals lacked awareness of the need to reduce or cease substance use and often acted impulsively, making continuity of care and follow-up especially difficult. Frequent disengagement, service avoidance, and premature discharge were identified as common obstacles, reinforcing the view that recovery depended heavily on insight, treatment acceptance, and sustained engagement with support.

#### HCP4.2: Family can’t provide care anymore.

Healthcare professionals noted that family support, although central to recovery, was not always available or sustainable. Some families experienced ongoing conflict, while others were constrained by demanding work commitments, financial difficulties, limited understanding of mental illness, or their own health problems, all of which reduced their capacity to provide consistent care. These accounts suggests that recovery could be significantly compromised when the family system itself was under strain or no longer able to fulfil a caregiving role.

*“Most psychiatric patients have no family support. When their parents or caregivers die, how are they supposed to survive? This becomes a major issue. Many become homeless or wander the streets.”*

*- HCP08*

#### HCP4.3: Community reluctance to reintegrate individuals with mental illness.

Healthcare professionals described community stigma and fear as significant barriers to recovery, particularly for individuals returning to the community following hospitalisation or those with histories of substance misuse or behaviours that had previously caused concern among community members. Reluctant to accept these individuals back, rooted in perceptions of unpredictable or risk, made social reintegration difficult even following treatment completion. Trust was especially challenging to rebuild where previous behaviours had caused harm within the community.

#### HCP4.4: Staff challenges in supporting patient recovery.

Healthcare professionals described significant organisational constraints that limited their ability to deliver recovery-oriented care, despite positive attitudes towards supporting patients. Staff shortages, heavy workloads, extensive administrative demands, and broad service responsibilities reduced opportunities for sustained mental health support and relationship-based care. General nurses and public health officers working in subdistrict hospitals also reported low confidence in managing mental health needs, particularly in the absence of specialist psychiatric training. The lack of psychiatrists within primary and community mental health services further restricted care options and clinical decision-making capacity.

*“Treatment orders from a psychiatrist carry more authority. Doctors, by definition, have more decision-making power. We (non-physician) are here to assist, but without a psychiatrist, we’re limited as we can’t make those decisions ourselves […]. Our voice isn’t as strong […].”*

*- HCP05*

#### JST4.1: Employment barriers for people with mental illness.

Participants reported that many employers were reluctant to hire individuals with mental health conditions, often perceiving mental illness as affecting work performance and reliability. Inconsistent attendance such as working for brief periods followed by repeated absence, were seen as economically disadvantageous, further limiting opportunities for stable employment. These barriers restricted not only financial independence but also social inclusion and personal confidence, given the close association between employment, dignity, purpose, and recovery.

#### JST4.2: Peer influence on relapse among people with substance use difficulties.

Participants described the social environment as a major influence on recovery from substance misuse, with peer and friendship networks seen as shaping everyday behaviours and decisions alongside family support. Recovery was considered particularly vulnerable to peer influence among young people, for whom returning to friendship groups associated with substance use was viewed as significantly increasing the risk of relapse and perpetuating harmful patterns of behaviour.

### Reflection and validation at feedback workshops

Feedback from staff and service users at subsequent workshops indicated that mental health service users, carers, and healthcare professionals generally endorsed the findings. No participants expressed disagreement or identified additional themes.

### Recovery Support Model for personal recovery

**Fig 2:**
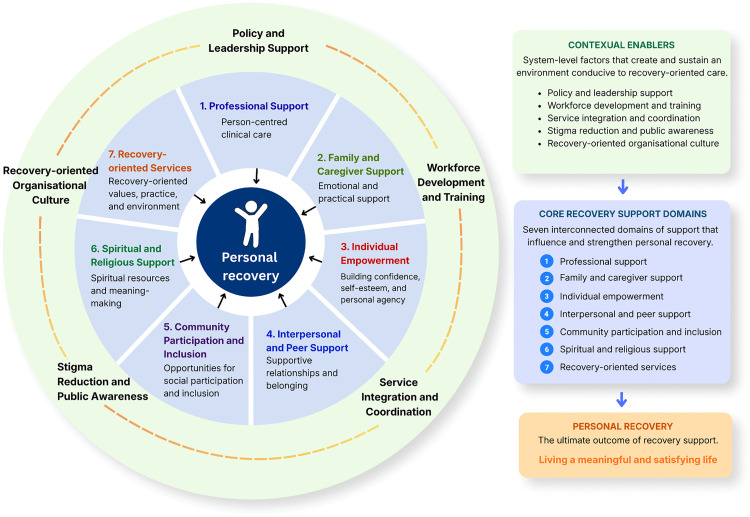
Recovery Support Model for personal recovery in Thai primary and community mental healthcare services.

Drawing on the in-depth analysis of interviews and feedback workshops, the research team developed a conceptual model of recovery support for people with mental illness and substance-related disorders in Thai community mental health settings (Fig 2). Findings from the interviews were presented to and validated by participants during feedback workshops prior to model development. The model components emerged directly from Theme 3 ‘Characteristics of a successful recovery journey’ and Theme 4 ‘Factors impeding recovery’, which explored recovery support from the perspectives of both service users and healthcare professionals. Although discussions were informed by the CHIME framework [29], the Brief INSPIRE measure [31], and the Recovery Self-Assessment for providers and family members [32], the model was not produced through deductive coding against these frameworks. Rather, it was developed inductively from participants’ experiences and perspectives, enabling the study to identify the aspects of recovery support considered most important within the Thai context. The model therefore reflects several contextual features that shape recovery support in Thailand, including strong family involvement, community interconnectedness, Buddhist influenced perspectives on acceptance, meaning, and merit making, and hierarchical relationships between service users and healthcare professionals. The model also recognises the important role of non-specialist providers such as village health volunteers in community mental healthcare.

The model comprises seven interconnected domains that support personal recovery among people experiencing mental illness: (1) professional support, (2) family and caregiver support, (3) individual empowerment, (4) interpersonal and peer support, (5) community participation and inclusion, (6) spiritual and religious support, (7) recovery-oriented services. These domains operate across individual, interpersonal, community, and service levels.

The practical implications of the Recovery Support Model are evident in its capacity to guide recovery-oriented practice, staff training, collaborative care planning, and service development within Thai primary and community mental healthcare settings. By identifying key domains of recovery support and the contextual enablers required for implementation, the model offers a culturally relevant framework for translating recovery principles into everyday clinical practice.

### Potential solutions for improving recovery-oriented care and practice

**Fig 3:**
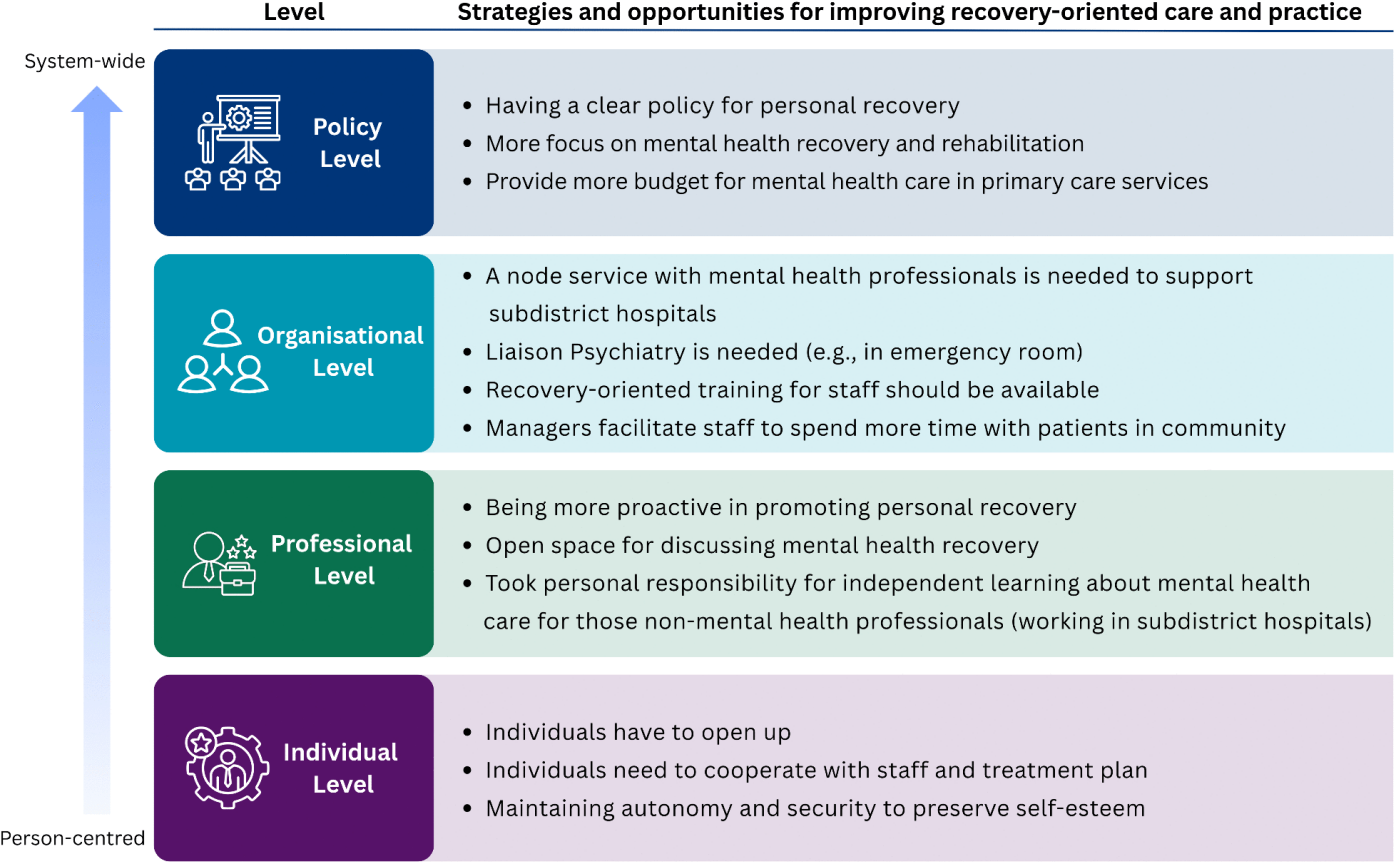
Strategies and opportunities for improving recovery-oriented care and practice.

During the interviews with participants, we discussed both challenging and supporting factors related to recovery-oriented care and practice. Based on these emergent examples of good practice, we summarised and conceptualised strategies and opportunities for improving recovery-oriented care and practice to better support the personal recovery of mental health service users within the study sites. These have been organised into different levels and are presented in Fig 3.

## Discussion

This study is the first to integrate the experiences and perspectives of mental health service users, carers, and multidisciplinary healthcare professionals to develop a contextually grounded and culturally situated understanding of personal recovery and recovery support in Thailand. It addresses an important gap in Thailand’s mental health landscape, where the concept of personal recovery is still evolving and has not yet been systematically embedded in clinical practice.

### Conceptions of mental health recovery

Findings indicated that Thai participants prioritised clinical and functional recovery before engaging with broader dimensions of personal recovery. Managing symptoms and resuming basic daily activities, such as self-care and employment were perceived as primary milestones of improvement [43]. Only after achieving these outcomes did participants begin to consider broader aspects of personal recovery, such as hope, meaning, and social connectedness. These findings are consistent with studies from Asia [44,45], Africa [46,47], and Europe [48], which suggest that recovery is often understood as both a return to a pre-illness state and as a transformative process, while mental illness is largely conceptualised within a biomedical framework. Nevertheless, the contextual understanding of personal recovery identified in this study was consistent with Anthony’s definition of recovery [49] and aligned with the CHIME framework [29]. Recovery was understood as a process beginning when individuals develop a more positive orientation, recognise the need for change, and establish future goals, enabling them to live independently, confidently, and with hope despite ongoing symptoms and challenges. Within the Thai context, personal recovery also emphasised both individual qualities, such as adaptability, resilience, and inner strength, and relational responsibilities, particularly the importance of avoiding repeated burden on others. This reflects broader values found in many Asian collectivist cultures, where individuals are encouraged to prioritise family responsibilities, social harmony, and collective wellbeing with personal actions shaped by social expectations and obligations [50,51].

### Attitudes towards mental health recovery

Findings indicated that attitudes towards recovery in Thailand were shaped by hierarchical relationships and challenging practice environments. Service users often deferred to healthcare professionals, reinforcing a power imbalance that may limit open dialogue and shared decision making and potentially constraining the development of person-centred and recovery-oriented care. While such dynamics may support engagement with treatment, they highlight the importance of promoting more balanced, collaborative relationships between service users and healthcare professionals [52]. Concurrently, healthcare professionals described their work as demanding and at times associated with risk and feelings of insecurity, particularly when supporting individuals with substance misuse in community settings. These pressures may foster more cautious and risk-focused approaches, where managing safety takes precedence over collaborative engagement, despite a general belief in the possibility of recovery.

These attitudes were further shaped by the low institutional priority afforded to mental health within the wider public health system. This is consistent with the Lancet Commission on global mental health and sustainable development and the Lancet Psychiatry Commission, both of which confirm that mental disorders continue to receive disproportionately low priority within global health systems [53,54].

### Characteristics of a successful recovery journey

Values such as compassion, family involvement, respect for authority, self-worth derived from helping others, and the influence of karma were deeply embedded in everyday life and shaped participants’ understanding of wellbeing and recovery within the Thai cultural context. Rooted in Buddhist teachings, compassion fosters empathy, acceptance, and kindness toward others, extending naturally to family relationships, where relatives often assume primary responsibility for emotional and practical support particularly where formal services are insufficient [55–58]. Within this cultural contexts, helping others carries significance beyond restoring self-worth or fostering social inclusion, providing care, volunteering, and supporting others are also understood as opportunities to make merit and fulfil important moral and spiritual responsibilities. Participation in meaningful activities therefore not only reduced rumination and provided distraction from distress, but also reinforced a sense of usefulness, interconnectedness, and spiritual purpose [13]. Concepts of karma and acceptance similarly appeared to shape how some individuals interpreted adversity and recovery, encouraging reflection, acceptance of difficulty, and gradual personal growth rather than positioning recovery solely as the elimination of symptoms [59].

### Factors impeding recovery

Recovery was constrained by multiple, overlapping barriers operating at individual (e.g., limited treatment engagement), family (e.g., insufficient support), service (e.g., limited guidance and follow up), and community (e.g., resistance to reintegration) levels. Consistent with other studies in Thailand [60,61], mental health care was perceived as largely focused on medication, with limited meaningful dialogue, highlighting a persistent gap between routine practice and recovery-oriented care. These challenges were particularly pronounced among individuals experiencing substance misuse, where relapse was shaped not only by individual behaviour but also by substance-using peer networks and broader social environments [62]. These issues cannot be viewed in isolation, as they are closely linked to systemic constraints including high workloads, limited mental health training, and insufficient resources [63]. Such structural limitations reduced opportunities for sustained therapeutic relationships and personalised care. Employment barriers and social stigma further restricted recovery highlighting that recovery is not solely an individual process but is shaped and often hindered by systemic, social, and service-level factors.

These factors were synthesised within the proposed Recovery Support Model, derived from the experiences of Thai service users, carers, and healthcare professionals. The model highlights factors that both support and impede recovery within the Thai sociocultural context. These included the influence of Buddhist beliefs and practices, characteristics of the Thai healthcare system and workforce, family and community support, and prevailing attitudes towards mental illness and recovery. Drawing on these insights, the model offers a culturally grounded framework for informing recovery-oriented practice and service development in Thailand.

### Strengths and limitations

This study included people with diverse mental conditions, including schizophrenia, depression, anxiety, stress, post-traumatic stress disorder, and alcohol dependence, along with their carers, and multidisciplinary healthcare staff, such as general nurses, mental health nurses, psychologists, and public health officers. Research settings were carefully selected to capture diversity in participants’ demographic backgrounds and mental health care experiences. Three subdistrict health-promoting hospitals were included: one in a rural area with a high prevalence of substance misuse; another covering both rural and urban areas with notable cases of substance misuse and mental illness; and a third situated in an urban district, primarily dealing with mental illness and suicide cases. This purposive selection allowed for a wide range of perspectives, ensuring that the data reflected a comprehensive understanding of mental health and substance misuse across different community contexts.

A PPI advisory team comprising a mental health nurse, psychiatrist, psychologist, peer support specialist/researcher, a person with lived experience of mental illness, and a carer provided an additional source of strength. Given that PPI, and particularly the active involvement of people with lived experience and carers as research partners remains relatively uncommon in Thai mental health research, this element enhanced the rigour and cultural relevance of the study. Their feedback on the Thai Global INSPIRE questionnaire and interview topic guides improved clarity and linguistic appropriateness reducing the risk of culturally insensitive phrasing and minimising potential stigma. Interview findings were subsequently reviewed and validated by participants during feedback workshops, supporting the accuracy and completeness of the data and ensuring key topics were adequately addressed.

Although carers were included alongside service users and healthcare professionals to capture multiple perspectives on recovery, the carer sample was relatively small. While valuable insights were obtained, a larger carer sample may have provided a broader understanding of family experiences and caregiving challenges within the recovery process. The limited participation of medical staff also represents a limitation, particularly given the hierarchical structure of the Thai healthcare system and the authority that psychiatrists and general practitioners hold in treatment decision-making. Contextual constraints significantly hindered their involvement: no psychiatrist was available at the study site, and a single general practitioner held responsibility for both mental health and physical health care, including emergency department duties. These competing demands created substantial time pressures, limiting their capacity to participate in the research.

### Implications for research, policy, and practice

Advancing recovery-oriented mental health care in Thailand requires systemic reform across multiple levels. At the individual level, recovery should move from a compliance-based model towards one that actively supports autonomy, participation, and dignity. Overemphasis on symptom control risks limiting personal agency and sustaining dependency on clinical services, highlighting the need for approaches that place service users at the centre of their own recovery.

At the professional and organisational level, the gap between policy aspirations and everyday practice remains substantial. Many staff working in subdistrict health-promoting hospitals and community settings have limited mental health training and often rely on self-directed learning, reflecting broader workforce capacity challenges and insufficient institutional support. Implementing recovery-oriented practice therefore requires sustained investment in workforce development through accessible training, ongoing supervision, and opportunities for reflective practice. Organisational support is equally essential, including protected time, manageable workloads, and sufficient staffing to enable meaningful engagement with service users beyond task-oriented care. Structural mechanisms such as liaison psychiatry services, node services that link community and specialist mental health care, and clear referral pathways may further support implementation. Without adequate workforce resources, managerial commitment, and supportive infrastructure, recovery-oriented care risks remaining a policy aspiration rather than becoming embedded in routine practice.

At the policy level, the absence of a clear national implementation framework for personal recovery contributes to fragmented service provision and inequitable access to care. Greater investment in rehabilitation, workforce development and community-based mental health infrastructure is needed to shift the system towards sustained, recovery-oriented care that is consistently available across settings.

Future research should examine how recovery principles can be implemented and sustained across levels of care, with priority given to participatory and longitudinal approaches capable of generating culturally grounded evidence to inform national mental health policy and reform in Thailand.

## Conclusion

This study highlights the need to transition from a predominantly clinical model of care to a more holistic, recovery-oriented approach within Thai community mental health services. Personal recovery involves not only clinical support but also the nurturing of personal agency, family involvement, and community engagement, supported by a well-trained and compassionate professionals. Promoting cross-sector collaboration, enhancing carers’ understanding through education, and integrating person-centred values into policy and service design can help create more hopeful, inclusive, and empowering environments for people living with mental illness and substance misuse in Thailand.

## Supporting information

S1 File Topic guides. (DOCX)

S2 File Quotes across themes and subthemes. (DOCX)
