# Supplementary material for: Views on mental health recovery in primary and community mental healthcare services in Thailand: A qualitative study
Source: PLoS One. 2026 Jul 20;21(7):e0353706. doi: 10.1371/journal.pone.0353706 (PMC13384306; doi:10.1371/journal.pone.0353706)
Supplement: S1 File — (DOCX) [file pone.0353706.s001.docx]

**Supplementary File 1**

**Topic guides**

***Staff topic guide***

Thank you for agreeing to this interview; I truly appreciate your time. We are interested in gathering your experiences of caring mental health patient in the community as well as your thoughts and perceptions about mental health recovery in your practice. The conversation is expected to last from half an hour and up to an hour.

**Warm up questions:**

- Years qualified
- How long been working in mental health care
- How long been working in community mental health care

**RECOVERY MEANING AND RECOVERY PRIORITY**

1. **What would mental health care look like in your setting?**

Prompts / Continuation Questions:

- What do you do for patients as part of your duty of care?
- What do you personally do to provide mental healthcare for your patients?
- What is the main priority or goal of your team/setting?
- What measures does your team/setting use to indicate the success of your care?

1. **What does the recovery from mental illness mean to you?**

Prompts / Continuation Questions:

- When I say the phrase “personal recovery” what thoughts immediately come to mind?
  - CHIME: Connectedness, Hope, Identity, Meaning and purpose, Empowerment
- How do you know if your patient gets recovered from mental illness?
- What do you think of this concept?
- Do you agree with this concept?
- Can you share your personal experience or give an example?

**3. What is your priority for your patient’s mental health recovery (quality of life: living a satisfying and hopeful life)?**

Prompts / Continuation Questions:

- CHIME: Connectedness, Hope, Identity, Meaning and purpose, Empowerment
- What do you think is the first step to achieving a good quality of life (or recovery)?
- **Patient factors:**
  - What kind of people can be recovered? / Who can be recovered? (people with mental illness, substance misuse)
- **Professional factors:**
  - Do you think (we) healthcare professionals play a role in helping? If so, how? (knowledge, attitudes)
- Organisational factors:
  - Do you think organisations/hospitals play a role in helping? If so, how? (policy, measure/indicators)

**RECOVERY SUPPORT**

**4. How do you think you are providing (recovery-oriented) care to support your patients’ recovery (quality of life)?**

Prompts / Continuation Questions:

- Questions/prompts adapted from the Recovery Self-Assessment for provider (O’Connell et al., 2005)
  1. **Life goals beyond symptom management**
     - You help patients to develop and plan for life goals beyond managing symptoms or staying stable (e.g., employment, education, physical fitness, connecting with family and friends, hobbies).
     - You actively help patients to get involved in non-mental health / addiction related activities, such as church groups, adult education, sports, or hobbies.
     - Your primary role is to assist patient with fulfilling his/her own goals and aspirations.
  2. **User involvement and recovery education**
     - You actively help patients find ways to give back to their community (i.e., volunteering, community services, neighbourhood watch / cleanup).
     - You have patients involved with facilitating staff trainings and education.
  3. **Diversity of treatment options**
     - You offer patients opportunities to discuss their spiritual and sexual needs and interests when they wish.
     - You actively introduce patients to persons in recovery who can serve as role models or mentors.
     - You actively connect patients with self-help, peer support, or consumer advocacy groups.
  4. **Rights and respect**
     - Patient can change their clinician or case manager if they wish.
     - Patients can easily access their treatment records if they wish.
     - Staff listen and respect the decision that patients make about their treatment and care.
  5. **Individually tailored services**
     - You regularly ask patients about their interests and the things they would like to do in the community.
     - You work hard to help patients to include people who are important to them in their recovery/treatment planning (such as family, friends, clergy, or an employer).
     - Staff has a primary role to assist a patient with fulfilling his/her own goals and aspirations.

**5. What do you see as the main support to facilitate your recovery-oriented care/practice?**

Prompts / Continuation Questions:

- Professional factors
- Organisational factors

**6. What do you see as the main challenges that prevent you from providing/practising (recovery-oriented) care?**

Prompts / Continuation Questions:

- Professional factors
- Organisational factors

**7. How could current mental health care be improved to better support your patient’s personal recovery (quality of life)?**

Prompts / Continuation Questions:

- HCPs factors: recovery-related knowledge and attitudes
- Organisational factors: policy, determining measure or indicator
- What staff’s characteristics and attitudes do you think will promote patient’s recovery
- Anything you think will support your recovery related knowledge, attitudes, and competencies in your practice to support your patient’s recovery?
- Do you think any recovery-oriented training is necessary for you or your team?

**CLOSING QUESTION**

- Anything else you would like to add?
- Anything you think I’m missing when we talk about mental health (recovery-oriented) care in your setting?

**THANK YOU FOR YOUR TIME**

**Topic guides**

**People with mental illness topic guide**

Thank you for agreeing to this interview; I truly appreciate your time. As discussed, we will focus on your experiences, thoughts, and perceptions on your mental health recovery journey. The conversation is expected to last from half an hour and up to an hour, but if you need a break or wish to stop at any point, please let me know, and I'll be happy to accommodate your needs.

Please note that a camera will be filming the interview (if participants consented). Occasionally, I may need to check it briefly to ensure everything is working correctly. This will not affect my attention—I'm still fully engaged and interested in what you have to say. The camera simply helps me ensure we capture all the important details.

To help you ease into the discussion, we’ll start with some introductory questions:

- Your age
- Your diagnosis
- The number of admissions you've had

**RECOVERY MEANING AND RECOVERY PRIORITY**

1. **Can you tell me about your experience of having mental illness and how this affects your life?**

Prompts / Continuation Questions:

- How did you feel when you were (first) diagnosed with mental illness? (Might specify diagnosis if applicable)
- Can you tell me if having mental illness impacts your life and how?

1. **What does the recovery from mental illness mean to you?**

Prompts / Continuation Questions:

- When I say the phrase “personal recovery” what thoughts immediately come to mind?
  - CHIME: Connectedness, Hope, Identity, Meaning and purpose, Empowerment
    - Using some of 20-item in the Global INSPIRE questionnaire
- How do you know if you get recovered from your mental illness?

1. **What is your priority for your mental health recovery (living a satisfying and hopeful life)?**

Prompts / Continuation Questions:

- Thinking of the questionnaire you have completed previously
  - Using some of 20-item in the Global INSPIRE questionnaire to guide patients

**RECOVERY SUPPORT**

1. **Can you tell me about your experience receiving community mental health care?**

Prompts / Continuation Questions:

- Good experiences
- Bad experiences
- What made the experiences either good or bad for you?
- Do you think it helps you get better?
  - Clinical improvement
  - Well-being
- Anything you’ve seen staff personally do?

1. **How do you think mental health care you have currently been receiving supports your personal recovery (to live satisfyingly and hopefully)?**

Prompts / Continuation Questions:

- Does your health professionals support your recovery in relation to CHIME (Connectedness, Hope, Identity, Meaning and purpose, Empowerment) (Guided by the Brief INSPIRE questionnaire)
  - Your worker helps you to feel supported by other people
  - Your worker helps you to have hopes and dreams for the future
  - Your worker helps you to feel good about yourself
  - Your worker helps you to do things that mean something to you
  - Your worker helps you to feel in control of your life

1. **How could current mental health care be improved to better support your personal recovery (to live satisfyingly and hopefully)?**

Prompts / Continuation Questions:

- What do you want staff to do to support your quality of life (personal recovery)?
- What do you want staff to do to support you to live satisfyingly and hopefully (personal recovery)?
- What staff’s characteristics and attitudes do you think will promote your recovery?
- Do you think recovery-oriented training is necessary for staff?

**CLOSING QUESTION**

Anything else you would like to add?

**THANK YOU FOR YOUR TIME**

I will be in touch with you soon so that you can review your film and select the sections you would like to include in the 30-minute trigger film that will be presented to staff and other service users at the co-design event.

**Topic guides**

***Carer topic guide***

Thank you for agreeing to this interview; I truly appreciate your time. As discussed, we will focus on your experiences, thoughts, and perceptions on mental health recovery journey of people under your care. The conversation is expected to last from half an hour and up to an hour, but if you need a break or wish to stop at any point, please let me know, and I'll be happy to accommodate your needs.

Please note that a camera will be filming the interview (if participants consented). Occasionally, I may need to check it briefly to ensure everything is working correctly. This will not affect my attention—I'm still fully engaged and interested in what you have to say. The camera simply helps me ensure we capture all the important details.

To help you ease into the discussion, we’ll start with some introductory questions:

- Your age
- Your relationship with people with mental illness
- How long you care for people with mental illness

**RECOVERY MEANING AND RECOVERY PRIORITY**

1. **Can you tell me about your experience of caring for people having mental illness?**

Prompts / Continuation Questions:

- Good experiences
- Any challenges?
- Do you have any support? Whom do you get support from?

1. **What does the recovery from mental illness mean to you?**

Prompts / Continuation Questions:

- When I say the phrase “personal recovery” what thoughts immediately come to mind?
  - CHIME: Connectedness, Hope, Identity, Meaning and purpose, Empowerment
- How do you know if your relative get recovered from mental illness?
- What do you think of this concept?
- Do you agree with this concept?
- Can you share your personal experience or give an example?

1. **What is your priority for your relative’s mental health recovery (living a satisfying and hopeful life)?**

Prompts / Continuation Questions:

- CHIME: Connectedness, Hope, Identity, Meaning and purpose, Empowerment
- What do you think is the first step to achieving a good quality of life (or recovery)?
- What kind of people can be recovered? / Who can be recovered? (people with mental illness, substance misuse)
- Who do you think play a role in helping with your relative’s recovery? (his/herself, friends, family, community, HCPs)

**RECOVERY SUPPORT**

1. **How do you think mental health care that people you have cared for are currently receiving supports their personal recovery (to live satisfyingly and hopefully)?**

Prompts / Continuation Questions:

- Does it support you in relation to CHIME (Connectedness, Hope, Identity, Meaning and purpose, Empowerment)?
- Questions/prompts guided by the Recovery self-assessment for family members (O’Connell et al., 2005)
  - **Life goals beyond symptom management (example questions below)**
    - Staff help my loved one to develop and plan for life goals beyond managing symptoms or staying stable (e.g., employment, education, physical fitness, connecting with family and friends, hobbies).
    - Staff work hard to help my loved one fulfil his/her personal goals.
    - Staff help my loved one to get involved in non-mental health related activities, such as church groups, adult education, sports, or hobbies.
  - **User involvement and recovery education**
    - Staff help my loved one to find ways to give back to the community (i.e., volunteering, community services, neighbourhood watch/cleanup).
    - My loved one is or can be involved in facilitating staff trainings and education.
  - **Diversity of treatment options**
    - Staff introduce my loved one to others in recovery who can serve as role models or mentors.
    - Staff connect my loved one with self-help, peer support, or consumer advocacy groups.
    - My loved one is given opportunities to discuss his/her spiritual and sexual needs and interests when he/her wishes.
  - **Rights and respect**
    - My loved one can change his/her clinician or case manager if he/she wants to.
    - My loved one can easily access his/her treatment records if he/she wishes.
    - Staff listen to my loved one and respect his/her decision about his/her treatment and care.
  - **Individually tailored services**
    - Staff regularly ask my loved one about his/her interest and the things he/she would like to do in the community.
    - Staff help my loved one to include people who are important to him/her in his/her recovery/treatment planning (such as family, friends, clergy, or an employer).
    - Staff listen, and respond, to my loved one’s cultural experiences, interests, and concerns.

1. **How do you think you are involved in supporting their personal recovery (living a satisfying and hopeful life)?**

Prompts / Continuation Questions:

- Questions/prompts guided by the Recovery self-assessment for family members (O’Connell et al., 2005)

1. Staff encourage me to have hope and high expectations for my loved one’s recovery.
2. Staff listen to me and respect my opinion about my loved one’s treatment and care.
3. Staff include me in my loved one’s recovery/ treatment planning.
4. **How could current mental health care be improved to better support personal recovery of people you care for?**

Prompts / Continuation Questions:

- What do you want staff to do to support your relative’s quality of life (personal recovery)?
- What staff’s characteristics and attitudes do you think will promote your relative recovery?
- What do you think helps enhance healthcare professionals' knowledge, attitudes, and skills in providing recovery-oriented care (to support a satisfying and hopeful life)?
- Do you think recovery-oriented training is necessary for staff?

**CLOSING QUESTION**

Anything else you would like to add?

**THANK YOU FOR YOUR TIME**

I will be in touch with you soon so that you can review your film and select the sections you would like to include in the 30-minute trigger film that will be presented to staff and other service users at the co-design event.
