# Supplementary material for: Views on mental health recovery in primary and community mental healthcare services in Thailand: A qualitative study
Source: PLoS One. 2026 Jul 20;21(7):e0353706. doi: 10.1371/journal.pone.0353706 (PMC13384306; doi:10.1371/journal.pone.0353706)
Supplement: S2 File — (DOCX) [file pone.0353706.s002.docx]

**Supplementary File 2**

**Quotes across themes and subthemes**

| **Themes** | **Subthemes** | **Quotes** |
| --- | --- | --- |
| **Theme 1: Conceptions of mental health recovery** | **Service users** | |
|  | Recovery begins with the hope of being free from clinical symptoms | - “Patients have hope. Healthcare professionals also have hope that they’ll recover and not feel anxious or have recurring symptoms. I think if care is given alongside treatment, patients will have hope. Whether it’s technology, medication, or future research, I have that belief.” **(Carer04)** |
|  | **Healthcare professionals** | |
|  | Clinical recovery must come first | - “During the acute or crisis phase, it’s really not manageable. It’s beyond our capacity at the subdistrict health promoting hospital. The patient must go through the proper treatment process (in psychiatric hospitals) first. Only then we can start having meaningful conversations.” **(HCP02)** |
|  | There is a strong alignment between functional and personal recovery | - “At the very least, they should be able to take care of their basic daily needs. If it goes better, they might even help with family tasks. What we hope for is that they can live independently. Then they can work, take care of themselves, start helping their family. Some can even return to normal functioning we’d call it full recovery.” **(HCP05)** |
|  | **Joint subthemes** | |
|  | Personal recovery could mean… | - “I want him to have good quality of life, better health, a better life. Like right now, it’s not that he can’t do anything.” **(Carer01)** - “Some things we also let her do on her own. For example, when going to see the doctor, we feel like she should go by herself sometimes. If we do everything for her, it will make her feel like she is severely ill. So, we try to let her connect and interact with other people as much as possible. It’s just part of life.” **(Carer04)** - “Feeling that I can control my life, that means I can steer things toward the good. I can live a good life going forward.” **(PwM01)** - “Patient can live their life happily, manage to integrate into the community, and participate in community activities, that’s what I’d call ‘getting better’.” **(HCP01)** - “In my opinion, it (personal recovery) happens because the patient starts to develop a better mindset on their own, a change in how they think. They begin to care for themselves, from previously not caring at all. Once they are able to mindfully reflect and think things through from within, they start to realise, 'I should be better. I could get better if I take my medication, if I stop doing certain things, let things go, engage in activities I enjoy, follow the doctor’s advice, take my medicine, attend appointments, and behave well with family.' So I think this internal mindset shift is what can help patients improve.” **(HCP02)** |
| **Theme 2: Attitudes towards mental health recovery** | **Service users** | |
|  | Staff are regarded with deference and authority | - “Some people, like relatives, fathers, mothers, or husbands, when they give advice, the patient won’t listen to them. But they’ll listen the doctor. Just like with my own children. When parents teach something, they don’t remember it. But when the teacher says something, they remember everything. It’s like that. If the doctor gives an explanation, and the patient hears it, like ‘You should do this and that,’ then they’ll follow it.” **(Carer05)** |
|  | **Healthcare professionals** |  |
|  | Working in mental health care is challenging | - “There was one time when I was on shift alone, and a psychiatric patient came in. He came and asked to kill me. At that moment, I had to stay calm and report it to the police (laughs). But that patient kept coming and going. The police know about him. But he still refuses to get treatment or rehabilitation.” **(HCP03)** - “The main challenge is all about psychiatric patients here (in the community) who have issues with substance misuse. That makes our work risky and dangerous in providing care, because we don’t know what if the patient recovers and returns (from jail or substance rehabilitation hospital), they might harm (us?) or what if they still hold resentment or anger toward us?” **(HCP01)** |
|  | Multi-sectoral collaboration and shared responsibility for mental health care | - “There have been a lot of changes already. In the early days, people still stigmatised those with mental health conditions. But over the past 2–3 years, there’s been more awareness and involvement from organisations beyond just public health, especially when it comes to patients with mental illness or drug-related issues in the community. The impact of these issues is now more visible in society. For instance, when someone becomes violent or severely unwell, more agencies are taking notice, and attitudes are shifting.” **(HCP05)** - “It’s no longer just ‘they’re mentally ill, they should be isolated.’ Now, everyone sees it as a shared responsibility to help patients access treatment as early as possible. One example is the SMI (Serious Mental Illness) initiative, where patients can now be brought in for treatment immediately, without it being illegal. This enables access to emergency psychiatric care at any hospital.” **(HCP05)** |
|  | Belief in the possibility of recovery for all | - “In my opinion, from what I’ve seen, patients with depression who have entered the treatment process can return to normal life. If they receive proper care, counselling, take some medication, and have a positive mindset, they can go back to studying and working as usual.” **(HCP02)** - “Compared to people with depression, drug addiction recovery is much harder to achieve. But it is possible. There have been successful cases, driven by motivation from themselves and support from people around them.” **(HCP02)** - “Regardless of which group of psychiatric patients they belong to, everyone has the chance to recover. It depends on the supportive factors. It is not just about medication. If someone stops taking meds, they can still recover. Or if someone has schizophrenia, even with the illness, if their family provides proper care and they take their medication and attend appointments, then all groups have the same chance.” **(HCP11)** |
|  | **Joint subthemes** | |
|  | Mental health receives limited attention and prioritisation | - “No one ever came. No one visits the patient. Not the doctor from XXX Hospital (district hospital), not the doctor from the health centre (subdistrict hospital). No one comes. Maybe they don’t see him (patient) as severe as a bedridden case. Most of the people they go visit are those who are completely dependent, bedridden. That’s who they go see.” **(Carer01)** - “In my opinion, (community) mental health services are still quite limited and underdeveloped. As I mentioned, many people experience both physical and psychological symptoms, but most people including health personnel tend to focus only on physical health. Yet mind and body are closely connected. If the mind is unwell, the body cannot function properly either. Right now, (community) mental health services are still minimal and not comprehensive.” **(HCP11)** |
| **Theme 3: Characteristics of a successful recovery journey** | **Service users** | |
|  | 'It’s more about how we carry ourselves' | - “When we’re in society, it depends on how we behave. It’s not like, just because they say we have schizophrenia, we act all crazy and live without care. If we do that, we won’t be able to be part of society, right? I didn’t think that way. I don’t carry it with me. I think of myself as normal, just like anyone else. […] Just because the doctor said I had a mental illness doesn’t mean I have to act like a patient. Just be normal, like before I got sick.” **(PwM03)** |
|  | Feeling loved and supported | - “The love from the people around me, their trust and confidence in me, made a big difference. You could feel it in how they treated you, like how siblings or nieces and nephews treat each other, and I could sense and receive those feelings. The result was that everything gradually improved, little by little.” **(PwM03)** - “Living with family means being happy together, not arguing, not scolding each other, and trying to avoid problems in the extended family.” **(PwM10)** |
|  | Preserving self-worth and contributing to others | - “Really, if you help others and do it with sincerity, anyone who has the heart to help others will feel happiness from it. It brings joy. It depends on the person and your intention too. If you genuinely want to help others, when you get to do it, it feels like you fulfilled that purpose. It makes you happy. It helps pass the time, which is good. Helping others and doing public work means you don’t have time for nonsense. You won’t be stuck alone.” **(PwM09)** |
|  | Staying connected with people and nature | - “My heart may be tired and weary, tired from certain things. Is that related to mental well-being? I would say I have to find something to support myself. Nowadays I raise dogs, raise mice, watch birds, and go into the forest to look for herbs, or see if I can collect mushrooms. That kind of thing. It makes me be with nature more.” **(PwM02)** - “I choose who I want to talk to. Even if work doesn’t progress, at least I can talk to someone. But I’d rather be with nature. It helps me feel better.” **(PwM02)** |
|  | Feeling accepted and treated like everyone else | - “[…] because the people close to me didn’t treat me differently or specially. They treated me the same as before, nothing really changed. I could still go out, do my own errands, and live my life without needing anyone to constantly look after me. It wasn’t as if I had to depend on anyone or be supervised all the time.” **(PwM03)** - “I sometimes see them as just like ordinary people. Don’t treat them differently. Don’t speak in a way that makes them feel separated. Some people say ‘crazy,’ but I think people with mental illness might be happier than ordinary people. […]” **(Carer04)** |
|  | Reducing or stopping alcohol consumption | - “If he (patient) could just stop drinking, that’s the only thing, he would probably get better. […] The biggest worry is alcohol. If he quits, he can manage his own life. If alcohol is out of the picture, then I’m not worried anymore.” **(Carer01)** - “They (family) also try to stop me from drinking. Around two or three in the afternoon, when I dress as if I’m going out to drink, my mum will ask what I want to eat and say, ‘Drink quickly and come back for dinner.’ In the past, if I went out drinking, I wouldn’t be home until midnight. But now, if I leave at two, I’m expected to be back by four in time for dinner. I can’t drink as much as before, maybe just four or five bottles, then I come back home, eat, take my medicine, and go to bed.” **(PwM08)** |
|  | Religious support as a source of recovery | - “When I go to temples, pay respects to the Buddha, light incense, and make a vow in front of the main Buddha statue, then pour water to dedicate merit on holy days, I feel fulfilled. I feel content because I’ve done good deeds. The bad karma that affected me seems to ease and lessen. […] Once I felt that the merit I made helped me, it softened things. The karma that struck me eased up. Things got better.” **(PwM04)** - “[…] whenever I have problems or anything, and I go to church and come back, it all fades. It really helps […] Like today, I just got back from church. It feels like recharging my battery. I come back full of energy, smiling at everything.” **(PwM09)** |
|  | **Healthcare professionals** | |
|  | 'It has to start with patients themselves' | - “I think it has to do with openness to accept help. Like I said, it depends on whether the patient wants to get better. If they want to be normal, it’s about whether they’re ready to open up. But even for psychiatric patients, if they open up, they could also recover.” **(HCP04)** - “It really comes from within the individual, triggered by encouragement and support from those around them. For example, in XXX district, which is considered a red zone (means a high prevalence of substance misuse), there are people who have truly succeeded in recovering. But those who fail to quit often lack mental strength. Initially, when they start using drugs, it may be due to lack of knowledge, awareness, or life goals. Once they get convinced, quitting becomes very difficult.” **(HCP02)** - “However, those who have a strong mindset and clear life goals, or who possess knowledge and awareness, usually avoid falling into drug addiction. For instance, some hill tribe youths do well in life, while others fall into the trap of drug use. It all comes down to knowledge, attitude, and awareness.” **(HCP02)** |
|  | Recovery requires collective community involvement | - “If the community doesn’t stigmatise patients who have experienced mental health issues and people (community) understand them. I think that’s the most crucial thing that support patients in overcoming their past mental health problems.” **(HCP01)** - “Some families have to ask for cooperation from the community because the patient regularly drinks alcohol. So, we ask the community not to sell it to them. It works quite well. The family then asks the local shops not to sell to the patient, saying the doctor forbids them from drinking. They have to use that as a reason that ‘the doctor absolutely does not allow it.’ And that work too.” **(HCP02)** - “Here in XXX district, we provide mental health training to village health volunteers and community leaders, things like how to recognise symptoms of relapse or early warning signs, so that people in the community can help monitor and, if needed, bring the patient to the hospital early on.” **(HCP05)** |
|  | Staff must possess recovery-oriented knowledge, attitudes, competencies and skills | - “First and foremost, healthcare staff must have knowledge because we need to understand how to assess patients. Having knowledge includes various skills like communication and how to approach patients. Good interpersonal skills are essential, and their demeanour should be trustworthy and compassionate. They need to understand that the patient is not in a normal state right now.” **(HCP02)** - “In the process we follow, we have to admit that communication skills to encourage and empower the patient are essential. We try to motivate them to keep fighting. For example, we share successful cases (in recovery), not revealing who they are, but explaining the situation, what they did, and how they improved. This helps patients see the input, process, and outcome, so they can compare it with themselves and their family, thinking, ‘Maybe I can do it like that.’” **(HCP02)** |
|  | **Joint subthemes** | |
|  | Family as a central factor in recovery | - “We also try to explain that there is no one else left in the family, it is just you. If you (relatives) do not take care of them (patients), then who will do it? Some relatives are almost ready to give up, saying things like, ‘Just let it go doctor (nurse), forget about it.’ But we tell them that it is not possible to just walk away. If the family does not step in, then who will? Sometimes we ask them to give it one more try, telling them that just take the patient to see the doctor, help manage their medication, just try once more. And in many families, once they get past that point, the patient begins to improve.” **(HCP02)** - “(Recovery should start from) the people in the house and those around him (patient), whether good or bad, the people in the house must not trigger him. They have to treat him with importance and give him opportunities (to do things he wants). […]” **(Carer01)** - “It’s the caregiver (family). Being close, attentive, supporting the treatment, and being with her (patient) so she doesn’t feel alone. Making everything feel normal.” **(Carer04)** - “I want to have a family (wife). I want someone to take care of me. Living alone like this, it’s quiet and lonely. It’s normal for men to feel that way, wanting a partner, someone to talk to and comfort you. But a partner who’s sincere, not one who’ll leave.” **(PwM01)** - “I think I’d like to have a girlfriend. I’m single now. If I had a partner, she could hold me back, tell me, ‘Don’t go out, just have dinner.’ I’ve been single for almost ten years. I have no one, go everywhere alone, watch movies alone. Then I start thinking, I’m getting old and still don’t have a life partner. Before, my girlfriend would stop me from going out. Sometimes she’d say, ‘Let’s go to the movies instead of drinking.’ At least she could get me to stop. These days, I haven’t gone out for a while. So if I had a girlfriend, I think it would help.” **(PwM08)** - “I think my family. They are just as important. With my husband, we talk openly. If something upsets us, we discuss it. We don’t keep things inside or let thoughts like ‘I’m going to do this or that’ build up. If we’re unsure about something, we ask. If we have a disagreement, even a small one, we sit down and talk it out the same evening. Arguing is normal. That’s what I practice — don’t hide anything, be transparent. If someone borrows money or takes on a debt, say it out loud. Family is very important, especially my husband.” **(PwM07)** |
|  | Peer support as a promising approach | - “[…] Because sometimes, we ourselves haven’t been in their shoes. Sometimes we may not fully understand the patients the way someone with lived experience can, as their peer.” **(HCP05)** - “It would provide another option for patients. Not everyone responds well to the same type of treatment. Peer support could be an alternative for people who prefer that kind of interaction. Some may not want to talk to a doctor but would rather talk to a peer. So, yes, if we had the opportunity and funding to hire, it would be great. It would also allow for more flexibility.” **(HCP05)** - “Compared to now, back then I really needed encouragement. Simply put, I needed someone to guide me. If there’s no one to guide you, your thoughts spiral. Small things become big things. People with this (mental) illness, if you ask what they need, it’s someone who understands emotions and the mind to help guide them.” **(PwM09)** - “Small things become big problems. That’s how it is. People like us need someone who understands the mind and emotions to support and guide us. As for me now, I’m okay. But I mean people who are newly diagnosed, like I was before, should have someone to talk to. That would be better than just going in, picking up meds, and leaving. I don’t understand the system, really.” **(PwM09)** |
| **Theme 4: Factors impeding recovery** | **Service users** | |
|  | Barriers that discourage recovery | - “When I first became unwell, it lasted for over a year, sometimes two, before I managed to see a healthcare professional. I was too embarrassed to sit and wait in the clinic because people kept walking past. I couldn’t bear it and would cry wherever I was. Eventually, I told my children I couldn’t take it anymore and needed to see the doctor. So I got dressed, drove to XXX Hospital, and handed in my card and paperwork.” **(PwM04)** - “They (patient) can communicate and follow instructions, but they are lazy, because the medicines they take are all sedatives. After taking them for a while, the sedatives make them feel sleepy.” **(Carer03)** - “The doctor gave me more medicine, but it was too much. After I took it, I just didn’t want to do anything. Like my partner said, it wasn’t that I thought I was better and stopped taking medicine. It was that the doctor gave me too much. I couldn’t even open my eyes. I was sleepy all the time. I didn’t do anything, just wanted to sleep all day. So, I stopped taking it. But once I stopped, I couldn’t sleep again and had to go see the doctor.” **(PwM03)** - “The doctor jokingly asked, ‘Oh, why didn’t you take the meds?’ I told her it made me too sleepy. I have things to do. If I just sleep all day, I can’t get anything done. The doctor said, ‘Well, you don’t really have to do anything. Sleeping all day is fine.’ That’s what she said, just like that. So, I didn’t say much, just let it go. Then, she told me, ‘If you don’t want to sleep too much, you can skip one of the four meds that cause drowsiness.’ So now I don’t take one of them. I actually have four psychiatric medications. I just take the remaining three. I stopped the fourth one because it made me sleepy all day. Now I sleep fine.” **(PwM03)** |
|  | Insufficient support for recovery | - “[…] I don’t even know where to start with him anymore. Because no one comes to guide us. No agency has ever come to ask. It’s like the municipality should be surveying homes, asking whether there are people with illness or disability. But this house? Nothing. We’ve never received anything. They say there’s help for disabled households, but we’ve never gotten anything.” **(Carer01)** - “Maybe they think we’re managing fine on our own. Maybe they see us as someone who can earn a living. Or maybe I’m not thinking that far that they might see him (patient) as just an alcoholic. I don’t want to think negatively like that. Maybe they think he can take care of himself. But I’ve seen other households with visually impaired people, I wonder why they get support. One family has a member with a leg disability, and the municipality even gave them a wheelchair. But my nephew has never received anything, even when they came to inspect, nothing at all.” **(Carer01)** - “Other than giving the medicine to take at home, we (carers) have to monitor ourselves. Like if this or that symptom happens, we have to know what to do. Sometimes I’ve learned that when he (patient) drinks a lot, he lacks nutrients or electrolytes. So I give him a Sponsor drink (mineral replacement). Sometimes things like that help him sweat, flush out waste, urinate more, and then the symptoms ease a bit. That means the symptoms are less intense. Sometimes he won’t have seizures if I can manage it. But if he drinks heavily, then it’s really risky. He’s at risk of having a seizure.” **(Carer01)** |
|  | Mental health care remains distant from recovery-oriented practice | - “I think there needs to be more conversation with patients and more attentive follow-up. I don’t think just going to the hospital and getting medication counts as recovery. There should be ongoing conversation, so you can build a connection and understand where the person stands, how they are today, how they were three months ago, and how they might be in the future. Right now, I understand that there are too few staff. Sometimes, with so little time for conversation, it’s hard to really understand what they need. Even though I’ve taken care of her (patient) for 20 years, I still don’t understand everything. Some things just arise day to day. Something I do might annoy her, and I wouldn’t know why. That’s why I say medical professionals should talk with patients more. But I understand it’s difficult with so few staff compared to the number of patients.” **(Carer04)** - “The doctor should have explained things more clearly, like helping me adjust based on my actual daily life. Not just say it without thinking. They should consider real life. You can’t just sleep all day and night. Sleeping too much gives you a headache and you don’t get anything done.” **(PwM03)** - “Speaking honestly, in terms of support, they only ask me questions during checkups. They don’t say much else. At XXX Hospital, they just say things like, ‘You need to try to think positively,’ but not much more than that. They just take my history. I have to find things out on my own, search on Google about how to reduce depression and manage the symptoms.” **(PwM07)** - “Like if every visit, someone would ask how we’re doing, how we cope with stress. Like the way you’re (interviewer) asking me now. If someone asked these questions at the hospital, I think it would be helpful. Because when you’re newly diagnosed, you’re very emotionally fragile.” **(PwM09)** |
|  | Inequality in healthcare service | - “They (disability centre) didn’t want someone like him (patient). I went to ask. He never went to get a disability card at the provincial hall. His older sister did go to register for the card. I also asked if someone like him, who has mental illness and visual impairment, could be accepted there. They said they had conditions. Like can he (patient) do this, can he take care of himself. When I thought about it, it felt like they are selective. So, I gave up. So, we just carry on living together. The nurse once even said to me, ‘Just keep looking after each other like this, one of you will die before the other.’ So, I’ve just endured it. I don’t know whether it will be me or him who goes first.” **(Carer01)** - “[…] The municipality came to do a survey. We didn’t get anything. Maybe they’re biased. I saw that the father of one of the staff who came to do the survey received support. And another neighbour got something too. They even asked me, ‘You’re not going to pick up the items from the municipality today?’ And I said, I didn’t get anything.” **(Carer01)** |
|  | Challenges in addressing alcohol misuse | - “[…] At first, even when his (patient’s) sight was gone, he still went out to do labour work. But the money never made it home. It never reached grandma. He would tell her they only gave him a little, but in truth he got paid. The employer gave him money, and he would spend it on alcohol. He drank three times a day. If he had money, he’d drink. He even paid people to go buy alcohol for him. Then the seizures got worse. So, I told him, no need to go out to work anymore. Since the money never made it to grandma anyway, better stay home.” **(Carer01)** - “Honestly, I’ve tried everything. Like he (patient) said he wanted to raise chickens. I bought chickens. Got the netting. Buy him the chicken foods. When the chickens were growing and almost ready to sell, I told him, ‘You could probably sell the chickens now.’ But then he wasn’t patient. He went and told a buyer to come during the time I wasn’t home. Sold them. People who know him well would know that if he gets all the money, it’ll be gone. It’ll be spent on alcohol. But some people know they need to give the money to his aunty. Some know, but others don’t. And it’s all down the bottle.” **(Carer01)** - “But if there’s an event, honestly, I don’t want any festivals to happen at all. New Year, Songkran, housewarmings, funerals. I don’t want to be invited. If he drinks, that’s it. Even if he drinks like others, someone like him is more sensitive. What do they call it? His awareness, his mood, he can’t control it. He has no self-regulation.” **(Carer01)** |
|  | **Healthcare professionals** | |
|  | Patients’ lack of engagement in their own recovery | - “And another thing is that many of these (mental health) patients don’t want to admit they have a mental health condition. There’s bias. So we need to work on opening their minds, give them knowledge so they can accept it. It takes time. Even after they know, they still deny it. Like someone with depression or a psychiatric disorder, when we ask, ‘What brings you here today?’ they’ll say, ‘I’m not sick.’ When it’s like that, we just focus on educating them.” **(HCP07)** - “Sometimes patients don’t take care of themselves at all. They don’t wash their clothes, and shower, or clean their house (laughs). Their house becomes messy and they don’t work or earn any income. So it's like they become or create a burden for their family members.” **(HCP02)** - “In the cases from XXX subdistrict, most of them were situations where the patients refused treatment. We haven't had any cases where the patient completed rehabilitation and recovery and then returned to normal life.” **(HCP03)** |
|  | Family can’t provide care anymore | - “To be honest, there are typically two things. First, relatives are not very interested in understanding the illness or the patient. When we ask them to accompany the patient to appointments or to pick up medication, they have issues with not having anyone to take them or needing to work. It comes down to a lack of readiness. This is a real issue.” **(HCP10)** - “Most psychiatric patients have no family support. When their parents or caregivers die, how are they supposed to survive? This becomes a major issue. Many become homeless or wander the streets.” **(HCP08)** - “In community settings, we tend to focus on both the patient and their family. Sometimes we try to speak with the relatives, but they do not show much interest. It depends on how ready the family is. That is based on my experience. Perhaps in other areas, families might be more committed and attentive, truly caring and understanding. But in most cases I have encountered, it is not like that. Most often, it involves confinement.” **(HCP10)** |
|  | Community reluctance to reintegrate individuals with mental health doesn’t want people with mental health living in the community | - “This particular patient had used substances and caused problems within the community, including domestic violence and theft. After legal proceedings and treatment, the plan was to reintegrate her into the community. However, there were strong objections from community members who did not want her to return. When the district hospital contacted us (subdistrict hospital), they said the community was quite hostile and asked me to speak with the community leader.” **(HCP13)** - “In this case, because the patient had hurt the younger person before, the younger person was unwilling to accept his return and was scared. So, the team went to talk to family members and community leaders, like the village head and municipal leader, to create shared understanding that this person had received treatment, and if he kept taking his medications and avoided alcohol, he could come back and live in the community.” **(HCP05)** |
|  | Staff challenges in supporting patient recovery | - “With the responsibilities and workload, the relationship between the staff and the amount of work is quite conflicting. The number of patients is increasing, but the number of staff remains the same. As a result, access to treatment has decreased.” **(HCP01)** - “Treatment orders from a psychiatrist carry more authority. Doctors, by definition, have more decision-making power. We are here to assist, but without a psychiatrist, we’re limited as we can’t make those decisions ourselves […]. Our voice isn’t as strong […]. There used to be a psychiatrist, back then the psychiatrist could go directly to the hospital director and request things, and the director would support them because they had the authority to raise issues and shape policy more effectively, especially being a specialist doctor.” **(HCP05)** - “Also, as I mentioned before, our work is not limited to one task. We have to care for general patients who come for treatment, including those with NCDs (non-communicable diseases) like diabetes, hypertension, and kidney problems. We also take care of children, such as giving vaccinations and school health services, and look after children aged zero to five who come to the health centre for immunisations. So our work really covers many areas. Besides that, we also have paperwork and meetings to handle. There are many different responsibilities, so sometimes we cannot fully focus on everything.” **(HCP02)** - “Honestly, our staff aren’t specially trained in psychiatry. In Thailand, nurses have to handle everything, from prenatal care all the way to elderly care. That’s just how it is. Because of this heavy workload, we sometimes overlook psychiatric patients.” **(HCP02)** - “The rest of the patients, those without significant symptoms, are usually prescribed the same medication as before. The doctor can’t possibly see all 70–80 patients in one session.” **(HCP08)** |
|  | **Joint subthemes** | |
|  | Employment barriers for people with mental illness | - “There was a patient who came to receive an injection from me. He worked as a day labourer, but could not keep the job for long. Because of his condition and other reasons, people did not accept him. Wherever he applied, they would not hire him. In the end, he helped clean a temple. The monks gave him food from alms. He could not get a job anywhere else.” **(HCP11)** - “No one would hire them for work, right? They’d say, 'this crazy guy, why bring them here?’” **(Carer03)** |
|  | Peer influence on relapse among people with substance use difficulties | - “It’s all about drug addict. If they go back to their old friends, they might be influenced (by their friends) and end up going back in the same cycle.” **(HCP01)** - “Another case […] it involved a young man who had similar drug problems. He was the nephew of a village health volunteer. […] Being a teenager, he had friends, drank alcohol, smoked, and used drugs. […] Two rounds of rehab did not help. He relapsed both times. Eventually, they changed the approach. He moved in with his father and that solved the problem. The aunt said he now lived and worked with his father, no longer mixed with bad friends, and stopped using drugs. So I think the surrounding environment matters. Not just family but also friends. A young person’s social environment affects them. If that can be changed, it leads to improvement.” **(HCP10)** |
